# Fluorinated triazoles as privileged potential candidates in drug development—focusing on their biological and pharmaceutical properties

**DOI:** 10.3389/fchem.2022.926723

**Published:** 2022-08-09

**Authors:** Ihsan Ullah, Muhammad Ilyas, Muhammad Omer, Muhammad Alamzeb, Adnan, Muhammad Sohail

**Affiliations:** ^1^ Institute of Chemical Sciences, University of Swat, Swat, Pakistan; ^2^ Department of Chemistry, University of Kotli, Kotli, Pakistan

**Keywords:** 1,2,3-traizoles, 1,2,4-triazoles, fluorinated, anticancer, antibacterial, antiviral, inhibitory, biological properties

## Abstract

Fluorinated heterocycles have attracted extensive attention not only in organic synthesis but also in pharmaceutical and medicinal sciences due to their enhanced biological activities than their non-fluorinated counterparts. Triazole is a simple five-membered heterocycle with three nitrogen atoms found in both natural and synthetic molecules that impart a broad spectrum of biological properties including but not limited to anticancer, antiproliferative, inhibitory, antiviral, antibacterial, antifungal, antiallergic, and antioxidant properties. In addition, incorporation of fluorine into triazole and its derivatives has been reported to enhance their pharmacological activity, making them promising drug candidates. This mini-review explores the current developments of backbone-fluorinated triazoles and functionalized fluorinated triazoles with established biological activities and pharmacological properties.

## 1 Introduction

Majority of naturally occurring therapeutic models contain heterocycle subunits in their structures. Among these, nitrogen heterocycles, their analogs and derivatives are the most abundant. Derivatization and hybrid drug design along with transposition are some of the excellent tools to minimize the manufacturing-cost involved and to enhance drug development to counter resistance (Aly et al., 2020; Eya’ane Meva et al., 2021; Kumar et al., 2021).

Triazole is an important constituent molecule that is widely distributed in nature and found in many essential biomolecules (Kharb et al., 2011). Triazoles exist in two possible structural forms, 1,2,3-triazoles and 1,2,4-triazoles, and both have potentially broad spectrum of pharmacological activities. Different types of activities from biological to materials chemistry have been broadly explored (Kumar et al., 2021; Li and Zhang 2022). Their importance is not only due to abundance but also because they are one of the core structural components in both natural and synthetic drugs and drug candidates that impart a huge variety of biological and pharmaceutical properties including anticancer, antioxidant, antidepressant, anti-inflammatory, antidiabetic, anti-HIV, antimicrobial, antiviral, antineoplastic, antimalarial, antihistaminic, antitubercular, anticoagulant, antibacterial, antiallergic as well as immunomodulatory agents, and anesthetic and enzyme inhibitors (Yadav et al., 2018; Nemallapudi et al., 2021).

Furthermore, some recently developed drugs, for example, alprazolam (anxiolytic agent), ribavirin (antiviral agent), itraconazole and fluconazole (antifungal agents), rizatriptan (antimigraine agent) are triazole ring-containing molecules (Koparir 2019; Tekintas et al., 2022).

Moreover, significance of fluorine in medicinal chemistry has now been established since its synthetic incorporation into a variety of organic compounds have a huge impact on the pharmacological properties, viz., membrane permeability, metabolic stability, lipophilicity, and binding affinity (Purser et al., 2008; Hu and Umemoto 2018). In comparison to their non-fluoroalkylated analogs, fluorine-containing compounds usually show superior biological and pharmacological activities (Reddy, 2015; Zhu et al., 2018). It has been established that proton substitution by fluorine in heterocycles can produce fruitful pharmacokinetic properties due to enhanced stability and lipophilicity. In addition, fluorine being the most electronegative element, and therefore, the C to F bond is significantly polarized, shows highest ionic character having smaller size compared to other halogens (Nobile et al., 2021). It has been established that replacement of the oxidizable C-H bond by a C-F bond has a key role that enhances the biological half-life of a drug thereby increasing its metabolic stability (Yadav et al., 2018).

A variety of fluorinated substituents like -CF_3_, -SCF_3_, -SF_5_, and -OCF_3_ regulate the pH of the parent molecule, totally shifting its biological properties to the desired direction (Kanduti et al., 2016).

The greater dipole moment of the C to F bond has a huge effect on the conformational behavior of fluorinated heterocycles. Fluorine has been therefore incorporated into drug molecules enormously, to produce dipole–dipole and charge–dipole interactions in addition to the remarkable advantages of the changed physicochemical behavior (Ali et al., 2013; Mei et al., 2020).

However, fluorine-containing molecules are not that much abundant in nature although found abundantly in the Earth’s crust. Therefore, incorporation of fluorine and fluoroalkyl groups has been one of the most successful tools to obtain all-important organofluorines.

In the 1990’s, just 2% of fluorine containing drugs were available in the market; this number has increased to 20%–30% by 2015 (Zhou et al., 2016). In 2020, 13 drugs containing fluorine/substituents have been approved by the FDA and were introduced in the pharmaceutical market (Yu et al., 2021).

It has been reported that the incorporation of fluorine into this small molecule—triazole—imparts a significant enhancement to their biological activities in comparison to their non-fluorinated counterparts (Langer et al., 2011; Ali et al., 2013), for instance, fluconazole and gemcitabine. Until now, modifications of the triazole moiety have proved to be highly effective with lesser toxicity and improved potency (Mohamad Saimi et al., 2021).

**FIGURE 1:**
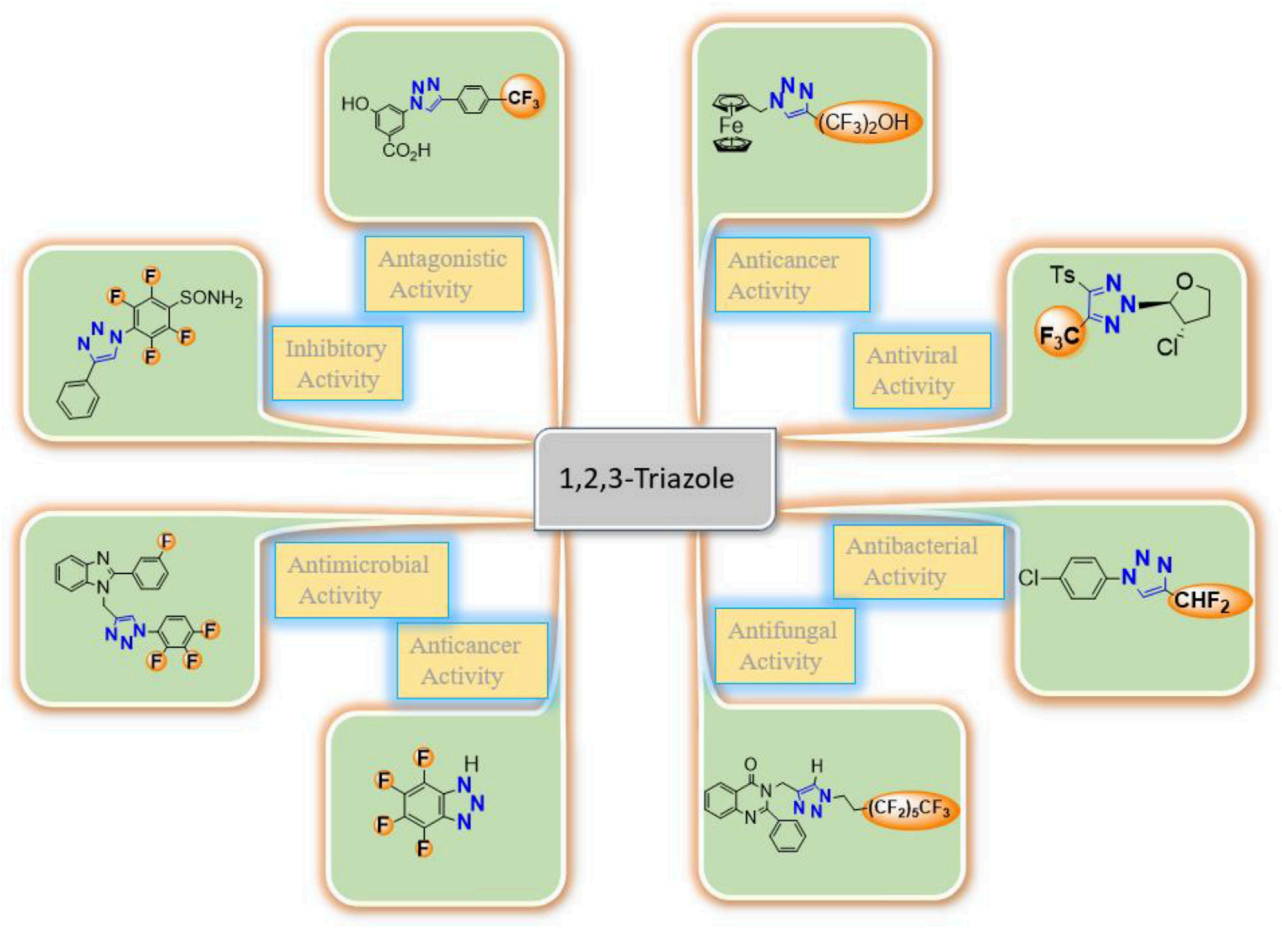
Representative examples of biologically important fluorinated 1,2,3-triazoles/analogs.

**FIGURE 2:**
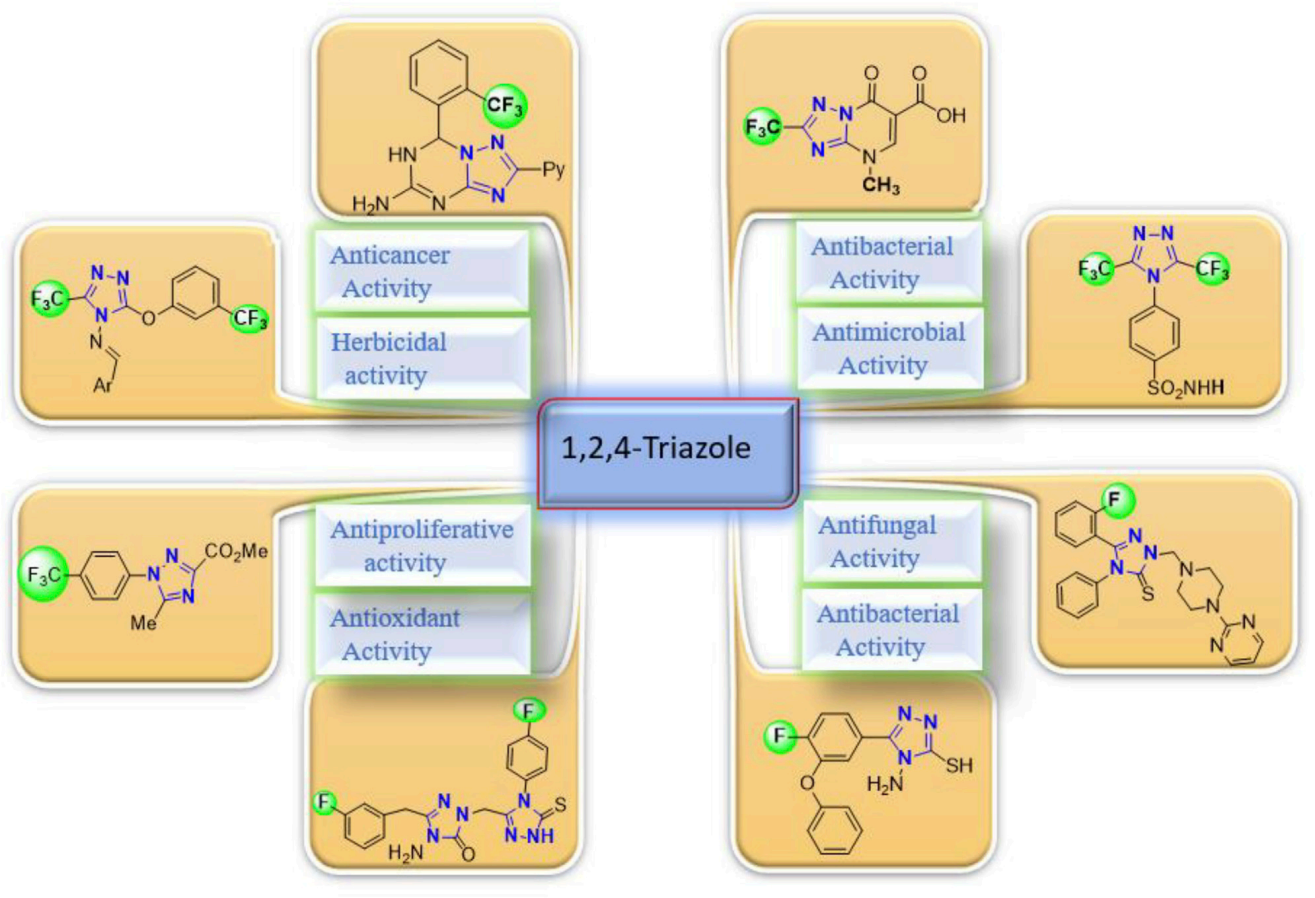
Representative examples of biologically important fluorinated 1,2,4-triazoles/analogs.

This mini-review highlights the significance of privileged backbone and/or functionalized fluorinated 1,2,3- and 1,2,4-triazoles that have been reported with high bioactivity, pharmacological activities, and/or drug-like properties. Some of the representative 1,2,3- and 1,2,4-triazoles with biological/pharmacological properties and structures are given in Figures 1, 2, respectively. The mini-review is aimed at summarizing backbone and/or functionalized fluorinated 1,2,3- and 1,2,4-triazoles with anticancer, antibacterial, antifungal, antiviral, antimicrobial, herbicidal, inhibitory, antioxidant, antagonistic, antimalarial, and anti-inflammatory properties. All the triazoles and/or analogs and hybrids are numbered properly, while their structures and names are given under respective activity along with reference in Supplementary Table S1. All the chemical structures were redrawn using ChemDraw. We claim this is the first ever study conducted in this direction for fluorinated triazoles.

### 1.1 Selected activities

#### 1.1.1 Anticancer activities

Cheng and coworkers have reported two fluorinated1,2,3-triazole analogs **1–2** (Supplementary Table S1) as potent HER2 tyrosine kinase inhibitors (Cheng et al., 2007). The compounds were synthesized by a simple two-step procedure and their *in vitro* bioactivity (of **1–2)** was investigated using MDAMB-453 (human breast cancer cells) tested by an MTT assay and phosphorylation inhibition of HER2 tyrosine kinase. The synthesized compounds have been confirmed by NMR, mass, and IR, and their binding modes have also been proposed after carrying out docking and molecular dynamics simulation studies. The fluorinated compounds were found to be the most active compared with other compounds with IC_50_ value for breast cancer cells, which has been reported to be 31.6 for **1** and 16.6 for **2**.

Dolzhenko and coworkers synthesized 12 new fluorinated 1,2,4-triazole analogs **3–14** (Supplementary Table S1) using a simple and effective three-step procedure and evaluated them for anticancer activity (Dolzhenko et al., 2008). Using different MDA-MB-231, HT-29, and A549 (breast, colon, and lung cancer cell lines, respectively), the fluorinated analogs were examined for antiproliferative and DHFR inhibition. It was revealed that eight having trifluoromethyl group at the para position was found the most potent against the breast cancer cell line with IC_50_ = 28 mM. Generally, the lung cancer cells were found more resistant for treatment with synthesized compounds, while the colon cancer cells were more susceptible. The position of fluorine-containing groups was found to show a major role in the anticancer activity of the reported fluorinated triazoles. The analogs were also tested for antiproliferative potency employing MTT assay bovine DHFR was used for DHFR inhibition. The pyridine ring directly attached to the hetero-fused triazole ring having fluoro aryl group, one of the prominent compounds that can act as a potent drug candidate.

Park and coworkers have reported novel perfluoroalkyl 1,2,3-triazole-appended-deoxyuridines **15–19** (Supplementary Table S1) (Park et al., 2010) and subsequently checked for their anticancer activity employing three cancer cell lines (PC-3, MDA-MB-231, ACHN; prostate, breast, and renal cancer cell lines, respectively). Two well-known anticancer drugs floxuridine and doxorubicin were used for comparison. The deoxyuridine derivative featuring an appended perfluorodecyl-substituted triazole unit exhibited prominent anticancer effects. The perfluorodecyltriazole unit played a significant part in cancer cell growth inhibition. The efficacy of compound **18** was found to be due to its inhibition of the key enzyme TS (thymidylate synthase). It was for the first time that perfluoroalkyltriazole-substituted nucleosides have been reported as medicinal applications.

Two different 1,2,3-triazole analogs **20–21** (Supplementary Table S1) were designed and synthesized by Peterson and Blagg (Peterson and Blagg 2010). Two breast cancer cell lines (SKBr-3 and MCF-7) were used to check their antiproliferative efficiency, and they displayed IC_50_ values above 50. Triazole-containing novobiocin analogs were synthesized by incorporation of the noviose sugar was incorporated into **20** and **21** to obtain triazole-containing novobiocin, their phenols were noviosylated and their growth inhibitory activities were also determined. However, these compounds have been reported to be moderately active.

Stefely and coworkers have reported one fluorinated triazole analog **22** (Supplementary Table S1) among a series of other compounds by Stefely et al. (2010) through CuAAC and yielded inhibitors of cancer cell growth. The trifluoromethyl-substituted compound showed greater than average potency against MCF-7 (breast carcinoma). The coumarin-bearing compound was effectively active.

In the same year (2010), a nucleoside-bearing novel fluorinated triazole analog **23** (Supplementary Table S1) was reported by Yu et al. (2010). The synthesized compound was prepared in better yield and its *in vitro* cytotoxicity tests against different human carcinoma including liver (HepG2), lung (A549), pulmonary (LAC), and cervical (Hela) was carried out using the MTT assay. Floxuridine was used as the reference compound. Compound **23** was found to be more active than the reference drug showing EC_50_ ranging from 9.6 to 10.98 l M. The nucleoside-bearing compound with fluoro aryl group at triazole ring showed enhanced biological activity and can serve as the lead compound. Almost similar, compound **24** (Supplementary Table S1) with nucleoside replaced by fused heterocycle, has also been reported by Kumar and coworkers using click chemistry technique by Anil Kumar et al. (2011). The triazole conjugate showed modest inhibitory activity (IC_50_ = 5.6–29.6 l M) for breast carcinoma (MDA-MB-361). Compound **24** showed 49% inhibition to cell proliferation of breast carcinoma. The data reported shows that fluorinated triazole conjugate showed much better activity than non-fluorinated triazole conjugates.

Maschke and coworkers have synthesized four different trifluoromethyl-substituted metallocene triazoles (M = Fe and Ru) **25–28** (Supplementary Table S1), and their biological potency was checked for cancer cells (Maschke et al., 2012). The cytotoxicity activity for the fluorinated triazoles was examined against MCF-7, HT-29, and PT-45 (breast, colon, and pancreas cancer cell lines, respectively), and IC_50_ values of 33 µm were found. As evident from the IC_50_, the cancer cell lines were moderately sensitive to novel reported compounds. It was found that metallocene moiety played a major role for cytotoxic potential, whereas the incorporation of trifluoromethyl groups enhances the lipophilicity due to improved cell membrane permeability. The solid-state structures were also determined by X-ray crystallography. Furthermore, successful bioconjugation was also carried out using amino acid L-leucine.

Two series of fluorinated 1,2,3-triazole analogs **29–34** (Supplementary Table S1) were designed and synthesized by Zou et al. (2012). All six compounds **29–34** showed good *in vitro* inhibitory activity against the cervical (HeLa) and hepatoma (Bel-7402) carcinoma employing MTT assay. Better selectivity was shown for HeLa cell lines and comparatively low activity against Bel-7402 cell was observed. Compound **32** displayed potential antitumor bioactivity of 46.22% against Bel-7402 cells at concentration of 10 μg/ml. Furthermore, para substitution was found to be more beneficial than ortho/meta substitution to the inhibition rate.

Duan and coworkers have reported two fluorinated novel triazole hybrids **35–36** (Supplementary Table S1) (Duan et al., 2013a) showed broad-spectrum and promising anticancer activity with IC_50_ values in the range of 0.73–11.61 µM. The compounds were tested against four different human carcinomas including gastric MGC-803, breast MCF-7, prostrate PC-3, and esophageal EC-109 using MTT assay method. Compound **35** has been reported to be more potent than 5-fluorouracil.

A number of novel urea hybrids of 1,2,3-triazoles were reported by Duan et al. (2013b) with two fluorinated triazole hybrids **37–38** (Supplementary Table S1). The fluorinated hybrids **37** and **38** gave a broad spectrum and remarkable anticancer activity with IC_50_ values in the range 1.62–20.84 µM and 0.76–13.55 µM, respectively. Both the compounds were tested against four different human cancer cell lines including gastric MGC-803, breast MCF-7, prostrate PC-3, and esophageal EC-109 using an MTT assay method. Steric hindrance on the N-atom was observed to be a crucial factor in antiproliferation from structure activity relationship (SAR) analysis. Fluorinated triazole hybrids, **37** and **38**, have been reported to be more efficient than non-fluorinated hybrids.

Avula et al. (2012) reported a number of 1,2,3-triazoles including four fluorinated compounds **39–42** (Supplementary Table S1). The compounds were prepared through a simple multi-step synthesis using 5-fluoroisatin as the starting material and subsequently screened against lung (A-549), cervical (HeLa), and prostate (DU-145) human carcinomas by an MTT assay to check their potential of cytotoxicity at 10.01 M concentration. The compounds **39–42** exhibited average cytotoxic efficiency against human reproductive organs carcinoma, while weaker cytotoxic effect was shown against different human cancer cells. Presence of fluorine significantly increased their efficiency against human reproductive organ carcinoma.

A number of fluorinated 1,2,3-triazoles-linked heterocyclic derivatives **43–50** (Supplementary Table S1) were synthesized by Kumbhare et al. (2015). Using breast (MCF-7), lung (A549), and skin (A375) carcinomas, the compounds were screened for their anticancer activity. FACS was used to check the efficacy of compounds and result demonstrated G2/M cell cycle arrest of MCF-7 cells. Apoptosis-inducing ability of compounds **43**, **44**, and **48** were tested against fundamental proteins NF-kB, survivin, CYP1A1, and ERK1/2, which help in cancer cell proliferation. The apoptotic character of these analogs was further revealed by enhancement in the activity of caspase-9 in MCF-7 cells. The study of the analogs on four selected carcinomas exhibited higher antitumor activity on MCF-7 breast carcinoma than others as evidenced by studies such as cell cycle arrest, cytotoxicity assay, and inhibitory activity on levels of expression of main proteins such as ERK1/2, NF-kB, survivin, and CYP1A1 that play an important part in estrogen-positive breast cancer cell proliferation. Compounds **43–50** along with standard positive control such as doxorubin (Doxo) and paclitaxel (Pacli) were compared. All the reported triazoles exhibited promising cytotoxicity with **43**, **44**, and **48** as the most active of all. On the basis of observed IC_50_ value, it was noticed that among all carcinomas, MCF-7 cells are most prone to triazoles. Compounds **43**, **44**, and **48** affect the expression of main proteins such as ERK1/2, NF-kB, and survivin that cause out-of-control cell proliferation.

Kurumurthy has synthesized a number of 1,2,3-triazole-tagged derivatives (Kurumurthy et al., 2014). Six novel fluorinated 1,2,3-triazole-tagged pyrazolo-pyridine compounds **51–56** (Supplementary Table S1) were also prepared and screened for their anticancer activity. The activity was checked against different cancer cell lines (Supplementary Table S1). But compounds **51** and **53** were found to be the most active.

Prima and colleagues have reported Fluorinated benzo-fused 1,2,3-triazoles (Prima et al., 2017) **57–60** (Supplementary Table S1). The synthesized triazoles were checked for their cytotoxicity toward the laryngeal epidermoid (Hep2) cancer cell lines showing cytotoxicity with IC_50_ of 2.2–26.4 μm and induced the cells apoptosis at concentrations C = 1–25 μm. Meanwhile, they were found to be non-toxic against normal cells. X-ray diffraction (XRD) confirmed the purity and authenticity of the compounds. A high-content screening method was applied by employing a main stain with Hoechst 33342 and propidium iodide to detect cytotoxicity that allowed distinction based on morphological criteria among live, apoptotic, and dead cells.

Wang and coworkers reported a series of 1,2,3-triazole derivatives (Wang et al., 2018). Among them, 11 fluorinated compounds were also prepared. The prepared triazoles were examined for the IC_50_ values against three carcinomas including lung (A549), liver (HepG2), and breast (MCF-7). The triazoles bearing aminophenoxy group with F-atom **61–71** (Supplementary Table S1) were found useful for the activity as compared with non-fluorinated counterparts. Moreover, replacement of CH3 group by CF_3_ group at 5-C position of triazole proved to be more active. The same trend was also found in the compounds **61–71**. The F-atom also showed to ameliorate the hydrophobicity and stability of aminophenoxy that reasoned for the better efficiency. Furthermore, fluoro-substituted aryl moiety also affected the cytotoxicity of triazoles, especially when present on C-4 position, they exhibited better activity, such as **63** and **70**.

Sayeed and coworkers have reported 1,2,3-triazole hybrids linked with imidazopyridine (Sayeed et al., 2018) and subsequently checked for their cytotoxicity. Three fluorinated compounds **72–74** (Supplementary Table S1) were also synthesized and were evaluated against four different carcinomas: lung (A549), prostate (DU-145), colon (HCT-116), and breast (MDA-MB 231) cancer cell lines. The triazoles showed better activity against all carcinoma. Colchicine and taxol were used as the standard reference. The IC_50_ values of the fluorinated compounds exhibited remarkable cytotoxicity in the range of 0.51–47.94 µM. The study demonstrated that the synthesis of imidazopyridine-linked-triazole conjugates as promising anticancer agents causing G2/M arrest and apoptosis-inducing ability.

A series of 1,2,3-triazole derivatives of melampomagnolide B (MMB) were reported by Janganati and coworkers through click chemistry protocol and checked against 60 different human carcinomas (Janganati et al., 2018). Among the synthesized derivatives, the fluorinated compound **75** (Supplementary Table S1) showed the best results for anticancer activity against all carcinoma cells and has been reported as lead compound for anticancer activity giving GI50 (half growth inhibition) values in the nano-molar ranging from 0.02 to 0.99 µM. EC50 values of 400 and 700 nm were exhibited by the lead compound **75** using two AML clinical samples. Compound **75** proved to be more effective than parthenolide as inhibitor of p65 phosphorylation in both hematological and solid tumor cell lines, indicating its capacity to inhibit the NF-κB pathway.

Wu and coworkers designed and synthesized 60 novel 1,2,3-triazole pharmacophores bearing allogibberic acid (Wu et al., 2018). Among the synthesized compounds, two fluorinated triazole compounds **76–77** (Supplementary Table S1) exhibited the best cytotoxicity results of all hybrid derivatives. The cytotoxicity was checked *in vitro* against different carcinomas including HL-60, A549, SMMC-7721, SW480, and MCF-7 (myeloid leukemia, lung, liver, colon, and breast, respectively) using taxol and cisplatin (DDP) as standard drugs. Both hybrids **76–77** exhibited selectively eight times more cytotoxicity than the standard cisplatin (DPP) toward MCF-7 and SW480. Furthermore, **76**—a fluorinated compound—was more potent than cisplatin (DDP) against all tested five tumor cell lines, with IC_50_ values in the range of 0.25–1.72 µM. Apoptosis and the cell cycle distribution experiments were also carried out for hybrid compound **76**.

Narsimha and coworkers reported a number of novel fluorinated 1,2,3-triazole derivatives **78–86** (Supplementary Table S1), and their *in vitro* anticancer activity was checked (Narsimha et al., 2020) in MCF-7 and HeLa (breast and cervical carcinoma cell lines) using an MTT assay. Most of the triazoles were observed to be moderately active against both carcinomas. Compound **79** and **80** showed best with IC_50_ values of 11.18 ± 1.01 to 33.15 ± 2.14 μM.

#### 1.1.2 Antibacterial activities

Maria S. Costa developed a series of new 1,2,3-triazole-4-carbaldehydes with N substituted by phenyl and carried out *in vitro* antimycobacterial activities (Costa et al., 2006). Among the series, **87–89** (Supplementary Table S1) were fluorinated derivatives. The synthesized compounds were tested against ATCC27294 (*Mycobacterium tuberculosis* H37RV strain), susceptible to both rifampin and isoniazide. The experiments were carried out using MABA (Microplate Alamar Blue Assay). The MIC (1 g/ml) (minimum inhibitory concentration) was found to prevent a color change from blue (no growth) to pink (growth). The compounds (**87–89**) were also confirmed by X-ray diffraction.

A number of fluorinated 1,2,4-triazole-fluoroquinolone analogs bearing carboxylic acids **90–94** (Supplementary Table S1 **)** were reported by Hamdy M. Abdel-Rahman (Abdel-Rahman et al., 2009). The reported analogs were tested against H37RV (*M. tuberculosis*) strain at concentrations of 6.25 1 g/ml. Screening was carried out using TAACF (*tuberculosis* antimicrobial acquisition and coordinating facility) in BACTEC12B medium using MABA (microplate Alamar Blue assay). Triazoles **90–94** exhibited moderate growth inhibition in the range of 26%–38%. The known lipophilicity factor of the fluoroquinolone played a significant role for penetration of the reported analogs through bacterial cells. A modified technique for disc diffusion was used initially to check the activity of analogs against *bacillus* cereus and *E. Coli* Gram-positive and Gram-negative bacteria, respectively, at *in vitro* growth inhibition. Compounds **90** and **93** exhibited better results against the tested microorganisms.

A novel clubbed [1,2,3] triazole-bearing fluoro-benzimidazoles were reported by Charansingh Gill to be remarkably potent for curing tuberculosis as H37RV strain inhibitors based on the preliminary results for **95–100** (Supplementary Table S1) (Gill et al., 2008). The compounds were assessed against *Escherichia coli*, *Pseudomonas aeruginosa*, *Staphylococcus aureus*, and *Salmonella* typhosa. From SAR studies, compounds **95, 96,** and **97** exhibited marked inhibition while **98, 99,** and **100** were moderately to least active. With comparative reference to gentamycin, fluorine may have played a crucial role for the better activity of the reported compounds. Initially, compound **95** bearing fluorine showed remarkable antimycobacterial potency of >96% inhibition at 6.25 mg concentration in comparison to non-fluorinated counterparts with less than 90% inhibition at the same concentration. Later on, fluorination was carried out to synthesize **96**, **97**, **98**, and **99** that exhibited promising results with >96% of inhibition at 6.25 mg concentration.

Havaldar and Patil prepared 1,2,4-triazole analogs with two fluorinated compounds **119** and **120** (Supplementary Table S1) (Havaldar and Patil 2008). Using the ditch plate technique at a concentration of 50 μg/ml, the reported compounds were checked against *E. coli*, *S. aureus*, and *Bacillus subtilis* for *in vitro* antibacterial activity. DMF was employed as solvent control, and nutrient agar was used as culture media.

Karthikeyan and coworkers synthesized a number of 1,2,4-triazole hybrids that were prepared utilizing 3-(2,4-dichloro-5-fluorophenyl)-4H1,2,4-triazole-3-thiol as starting material, with two fluorinated compounds **103–110** (Supplementary Table S1) among (Karthikeyan 2009). Disc diffusion technique was employed for screening of the antibacterial activity of reported triazoles against several bacterial strains including *E. coli*, *Klebsiella pneumoniae*, *P. aeruginosa*, *S. aureus*, and *Streptococcus pyogenes*. Compounds **103**, **105**, **106**, **107**, and **110** showed better results against all tested bacterial strains. Compounds **104**, **108**, and **109** were less active. Ciprofloxacin was employed as the reference drug. The highest dilution (lowest concentration) needed to inhibit bacterial growth was regarded as MIC (minimum inhibitory concentration).

A number of 1,2,3-triazole derivatives bearing pyrrolopyrimidine moieties were reported by Shiva Raju et al. (2019). Compounds **111–112** (Supplementary Table S1) were fluorinated and showed the best antimycobacterial activity of all the reported compounds. Evaluation was carried out against *M. tuberculosis* H37RV strain. The MIC (minimum inhibitory concentration) for compound **112** was found to be 0.78 μg/ml.

Mani Chandrika and coworkers have developed a series of novel 1,2,3-triazol derivatives bearing quinazolines moieties with a group of fluorinated compounds 113–118 (Supplementary Table S1) (Mani Chandrika et al., 2010). The compounds 113–118 were found potentially antimicrobial after *in vitro* screening against different Gram-positive (*Bacillus subtilis*, *S. aureus*, and *S. epidermidis*) and Gram-negative (*E. coli* and *P. aeruginosa*) strains by dissolving them in acetone. All compounds except **115** were moderately active.

Marepu and coworkers synthesized pyrido-fused N-arylated-1,2,3-triazole by regioselective application of Buchwald’s strategy (Marepu et al., 2018) with one of the compounds as fluorinated **119** (Supplementary Table S1). Compound **119** was then found to be a potential antimicrobial agent. *E. coli* (Gram-negative *E. coli*) and *B. subtilis* (Gram-positive *Bacillus* subtilis) were employed for the purpose, and 119 exhibited equivalent activity to that of streptomycin against the latter strain. However, **119** did not yield better results for the former strain in comparison to antibiotic streptomycin.

A fluorinated 1,2,4-triazole derivative **120** (Supplementary Table S1) was reported by Rezki et al. (2016). *In vitro* antibacterial and antimicrobial screening was carried out for 120 using three Gram-positive (*S. pneumoniae*, *Bacillus subtilis*, and *S. aureus*), three Gram-negative (*P. aeruginosa*, *E. coli*, and *Klebsiella pneumoniae*) bacterial strains, and two fungi (*Candida albicans* and *Aspergillus fumigatus*) by employing broth dilution method. With MIC of 16 lg/mL, compound **120** was found the most potent for Gram-negative bacterial strains.

Govindaiah and coworkers have synthesized a fluorinated 1,2,3-triazole derivatives bearing benzhydrylpiperazine moiety **121** (Supplementary Table S1) (Govindaiah et al., 2018). Using agar well diffusion method on *E. Coli* and *S. aureus*, antibacterial screening was carried out for compound **121**. Ciprofloxacin was employed as the reference drug against both negative and positive strains. The docking energy of 22.498 in comparison to the reference drug Ciprofloxacin was observed for the fluorinated **121**. Excellent inhibitory activity (zone of inhibition 16.45 and 15.63 mm, 16.15 mm) was observed for Gram-positive and Gram-negative bacterial strains, respectively, by **121**.

Two fluorinated 1,2,3-triazole derivatives **122–123** (Supplementary Table S1) were reported by Narsimha and coworkers (Narsimha et al., 2020), and antibacterial screening was performed. Stander broth micro-dilution technique was employed for antibacterial screening against three Gram-positive strains. Compounds **122** and **123** were tested against *S. epidermidis*, *S. aureus*, and *Bacillus subtilis* strains. Enhanced activity was shown for **122** and **123** owing to mono-fluorination at ortho/para positions of phenoxy group. In comparison to different references, **123** was found four times more effective against *Bacillus subtilis* (MIC = 1.56 ± 0.72 μg/ml compared to streptomycin MIC = 6.25 ± 0.21 μg/ml), two times more effective against *S. aureus* (MIC = 3.12 ± 0.85 μg/ml compared to streptomycin MIC = 6.25 ± 0.18 μg/ml), and equally effective against *S. epidermidis* (MIC = 3.12 ± 0.65 μg/ml with reference to streptomycin MIC = 3.12 ± 0.25 μg/ml).

Using CuAAC click approach, Deswal and coworkers reported one fluorinated 1,2,3-triazole derivative **124** (Supplementary Table S1) (Deswal et al., 2020), and it was subsequently tested for *in vitro* antibacterial activity against two Gram-positive (*Bacillus subtilis* MTCC 441, *S. epidermidis* MTCC 6880) and two Gram-negative (*P. aeruginosa* MTCC 424 and *E. coli* MTCC 16521) bacterial strains. Using Ciprofloxacin as the reference, the serial dilution method was employed. Compound **124** exhibited comparable potency, MIC value of 0.0047 μmol/ml, in comparison to the reference used.

Venugopola and coworkers reported fluorinated 1,2,4-triazole derivative **125** (Supplementary Table S1) (Venugopala et al., 2020). Compound **125** exhibited remarkable anti-TB activity against H37Rv and MDR strains of MTB at 5.5 μg/ml and 11 μg/ml, respectively. Of the four triazoles examined for their anti-TB activity, the best results were shown by **125** against H37Rv and MDR strains of MTB at 5.5 and 11 μg/ml, respectively.

Kosikowska and coworkers reported fluorinated 1,2,4-triazole analogs scaffold **126–131** (Supplementary Table S1) (Kosikowska et al., 2020). *In vitro* antibacterial activity was performed using six reference Gram-positive strains, eight MSSA clinical isolates, and eight MRSA clinical isolates. Minimal inhibitory concentration (MIC) was the basis for antibacterial screening, and they were observed in the range of 7.82–31.25 μg/ml for **126–131**.

Four fluorinated 1,2,4-triazole analogs **132–135** (Supplementary Table S1) were reported by Zeinab Muhammad and coworkers (Muhammad et al., 2021). Employing inhibition zone (IZ) method, **132–135** were evaluated for *in vitro* antibacterial activities against three Gram-positive (*Bacillus subtilis*, *S. aureus*, and *Enterococcus faecalis*) and three Gram-negative (*Proteus vulgaris*, *E. coli*, and *Enterobacter cloacae*) bacterial strains. As a standard Gentamycin was used good to moderate activities were exhibited.

#### 1.1.3 Antifungal activities

Karthikeyan and coworkers reported a series of derivatized fluorinated 1,2,4-triazoles **136–143** (Supplementary Table S1) (Karthikeyan, 2009). Antifungal screening of compounds **136–143** was performed using agar diffusion method on different strains of fungi including *Aspergillus fumigatus*, *Aspergillus niger*, *Candida albicans*, and *Penicillium marneffei*. Compounds **138** and **143** were found to exhibit the best activity against all tested strains of fungi. The reference drug used for comparison was Amphotericin B. The highest dilution (lowest concentration) needed to capture the growth of fungus was defined as MIC (minimum inhibitory concentration).

Marepu and coworkers synthesized 1,2,3-triazole analogs fused with pyridine by employing Buchwald’s strategy (Marepu et al., 2018). One of the compounds was fluorinated **144** (Supplementary Table S1) that was screened for antifungal activity. Compound **144** was found to be potentially useful against the two fungal strains used, *Fusarium ricini* and *Fusarium oxysporum*, comparable to the positive control mancozeb. Using fungal culture plates (at 0, 25, 50, 75, and 100 loadings), the MIC (minimum inhibitory concentration) was estimated.

A series of quinazoline-linked 1,2,3-triazole derivatives **145–150** (Supplementary Table S1) by Chandrika and coworkers (Mani Chandrika et al., 2010). Using agar cup diffusion method, *in vitro* antifungal activity was performed for **145–150** using different strains including MTCC 227 (*Candida albicans*), MTCC 36 (*S. cerevisiae*), MTCC 262 (*Rhizopus oryzae*), MTCC 1344 (*Aspergillus niger*), MTCC 277 (*Aspergillus flavus*), and NCIM 3462 (*Candida rugosa*). Among all compounds, **145** and **149** exhibited excellent performance at maximum concentration of 100 mg/ml. Compound **147** was found inactive against all fungal strains except for C. albicans. However, **149** was found to be the most potent antifungal agent.

A novel 1,2,4-triazole-bearing fluoro aryl group **151** (Supplementary Table S1) was reported by Yang and coworkers (Yang et al., 2015). Using mycelial growth rate method against five common pathogens, *in vitro* antifungal activities was performed. Compound **151** was found to show promising antifungal activity against *Alternaria solani*, *Cercospora arachidicola*, *Fusarium oxysporum*, *Gibberella zeae*, and *Physalospora piricola*. The inhibition rates for **151** against *Physalospora piricola* reached 100% at 50 gg/mL, while for other strains, it reached more than 90% at 50 gg/mL.

Four fluorinated 1,2,4-triazole-bearing piperazine **152–155** were reported by Zang and coworkers (Supplementary Table S1) through Mannich reaction (Zhang et al., 2016). Compounds **152–155** were screened for antifungal activity through mycelium growth rate at concentrations of 50 mg/ml against *Alternaria solani Sorauer*, *Cercospora arachidicola*, *Fusarium oxysporum*, *Gibberella sanbinetti*, *Physalospora piricola*, and *Rhizoctonia cerealis*. It was observed that maximum growth inhibitory activity was achieved at 50 mg/205 ml. Furthermore, for some strains, the compounds were more effective than the control Triadimef.

4-trifluoromethylbenzoate-linked 1,2,3-triazole **156–157** (Supplementary Table S1) were synthesized under aqueous conditions by Deswal et al. (2019). By employing standard serial dilution method, **156–157** were checked for potency against two fungal strains *Aspergillus niger* and *Candida albicans* (MTCC 8189 and 227, respectively). The prepared compounds were confirmed by X-ray crystallographic data (XRD). Good antifungal potency was observed for **156–157** as comparable to reference drugs ciprofloxacin and fluconazole.

In 2020, Deswal and coworkers have also reported indolinone-linked 1,2,3-triazole through click reaction (Deswal et al., 2020). Compound **158** (Supplementary Table S1) was fluorinated and screened for antifungal potency. Fluconazole was employed as a reference drug, and a MIC (in µmol/mL) was used. Compound **158** was tested against two different strains as mentioned above using SSDM (standard serial dilution method).

#### 1.1.4 Antiproliferative activity

Wang and coworkers have reported 1,2,4-triazoles as potent antiproliferative agents (Wang et al., 2011). Compounds **159–162** (Supplementary Table S1) were the fluorinated compounds and exhibited better inhibitory potency against a panel of human carcinoma *in vitro*, including lung cancer cells (NCI-H226), nasopharyngeal cancer cells (NPC-TW01), and T-cell leukemia (Jurkat) cells. The GI50 values obtained were 11.7 l M (for lung carcinoma), 15.2 l M (nasopharyngeal carcinoma), and 8.70 l M (leukemia), and therefore, demonstrated 50% decrease in cell growth in comparison to the vehicle.

#### 1.1.5 Antiviral activities

Wu and coworkers prepared fluorinated β-D-nucleoside-linked 1,2,3-triazole analog **163** (Supplementary Table S1) (Wu et al., 2013). The compound was observed to be a potent anti-HIV-1 having no cytotoxic effect at peak test concentration up to 25 µM. Compound **163** exhibited a nanomolar-level anti-HIV-1 activity (EC50 = 0.09 µM) that equals the standard reference AZT did (EC50 = 0.084 µM). Therefore, it is a remarkably potential candidate for future development to novel NRTIs (nucleoside reverse transcriptase inhibitors) for treatment of HIV-1 infection.

Biliavska and coworkers synthesized fluorinated 1,2,3-triazole **164** (Supplementary Table S1) (Biliavska et al., 2017) and reported them as novel promising chemotherapeutic agents. Using MTT test at concentrations of 50 μg/ml, it was observed that **164** suppressed HSV-1/US reproduction by 50%. PCR and cytomorphological methods in addition to MTT were used to identify infected cells containing specific virus inclusion. Infected cells were placed in the growth medium for treatment after virus adsorption at non-toxic concentrations. The EC50 (half maximal effective concentration) was estimated as 50% induction concentration of its maximal effectiveness.

A number of nucleoside-linked 1,2,3-triazole analogs having one fluorinated analog **165** (Supplementary Table S1) were prepared by Liu and coworkers (Liu et al., 2018). Compound **165** was subsequently checked for *in vitro* anti-HBV efficiency. Pronounced inhibition of HBV replicon at concentration of 5.0 µM was observed. The results are comparable for efficient inhibition of wild-type and lamivudine-resistant HBV-DNA replication in a time- and dose-dependent manner to that of 3TC at 20 µM. *In vivo* inhibition was observed as, the replication of DHBV-DNA in serum and liver effectively at a dose of 1 mg/kg/day.

Karypidou and coworkers synthesized a novel library of fused 1,2,3-triazole analogs containing two fluorinated compounds **166–167** (Supplementary Table S1) (Karypidou et al., 2018). They were screened against a coronavirus (229E) in HEL cells using reference compound UDA (Urtica dioica agglutinin). Compound **166** with EC50 = 8.95 µM proved to be the most potent antiviral agent.

#### 1.1.6 Antimicrobial activities

A novel clubbed [1,2,3] triazole-bearing fluoro-benzimidazoles **168–173** were reported by Gill and coworkers to be remarkably potent for antimicrobial activity (Supplementary Table S1) (Gill et al., 2008). The compounds were assessed against *E. coli*, *P. aeruginosa*, *S. aureus*, and *salmonella typhosa*. From SAR studies, compounds **168**, **170** and **172** exhibited marked inhibition while **169**, **171**, and **173** were moderately to least active. Comparative to reference gentamycin, fluorine may have played a crucial role for the better activity of reported compounds. Initially, compound **168** bearing fluorine showed promising antimycobacterial activity of >96% inhibition at 6.25 mg concentration in comparison to non-fluorinated counterparts with less than 90% inhibition at the same concentration. Later on, fluorination was carried out to synthesize **168**, **169**, **170**, and **172** that exhibited promising results with >96% of inhibition at 6.25 mg concentration.

Faidallah and coworkers have reported four trifluoromethylated 1,2,4-triazole analogs **174–177** (Supplementary Table S1) as promising antimicrobial agents by Faidallah et al. (2011). **174–177** were active for *in vitro* antimicrobial and antifungal activity against *E. coli*, *S. aureus*, *A. niger* and *C. albicans*. Furthermore, **174–177** also showed mild antidiabetic activity.

Yadav and coworkers have developed 1,2,3-triazole hybrids including one fluorinated compound **178** (Supplementary Table S1) through click reaction catalyzed by cellulose supported on copper nanoparticles (Yadav et al., 2018). Using serial dilution method **178** was tested against *B. subtilis* and *S. epidermidis* (Gram-positive bacteria), *E. coli* and *P. aeruginosa* (Gram-negative bacteria) and two fungal strains *A. niger* and *C. albicans*. For antibacterial analysis Ciprofloxacin and for antifungal analysis Fluconazole were employed as reference drugs. XRD analysis confirmed the self-assembling behavior of **178**. Docking studies revealed several non-covalent interactions of **178** with DNA topoisomerase. Molecular dynamics studies were also carried out to understand the dynamics of ligands interactions.

#### 1.1.7 Herbicidal activities

Zhang and coworkers synthesized novel fluorinated 1,2,4-triazole derivative **179** (Supplementary Table S1) through a multistep reaction (Zhang and Shi 2014). Initially, at concentration of 100 mg/L, the compounds exhibited inhibition of 79.5% against *B. campestris* L. Further **179** was tested against oil rape and barnyard grass, at different concentrations of 100 and 10 mg/L and gave weak herbicidal activity against *Echinochloa crus-galli*.

Liu and coworkers synthesized six novel fluorinated 1,2,4-triazole derivatives **180–185** (Supplementary Table S1) through microwave irradiation (Liu et al., 2013). At concentration of 100 ppm the synthesized compounds were tried for *in vivo* herbicidal activity against *B. campestris* and *E. crus-galli*. **180–185** remarkably inhibited the growth of B. campestris effectively from 66%–89% and were not effective against *E. crus-galli*. The studies were carried out using cup tests.

Zhang and coworkers have reported fluorinated 1,2,4-triazole derivatives bearing thione **186–189** (Supplementary Table S1) through Mannich reaction (Zhang et al., 2016). At concentrations of 100 mg/231 ml against *B. campestris* and *E. crus-galli*, **186–189** exhibited weak herbicidal activity. Tests were conducted *in vivo* through rape root method for *Brassica campestris* and barnyard grass cup method for *E. crus-galli*. The compounds were weakly active in comparison to the standard commercially available herbicide chlorsulfuron.

#### 1.1.8 Inhibitory activities

Wang and coworkers synthesized N-arylated-1,2,4-triazole derivatives. Among the series of compounds (Wang et al., 2011). **190–193** (Supplementary Table S1) were the fluorinated compounds and exhibited better inhibitory potency against a panel of human carcinoma *in vitro*, including lung cancer cells (NCI-H226), nasopharyngeal cancer cells (NPC-TW01), and T-cell Leukemia (Jurkat) cells. The GI50 values obtained were 11.7 l M (for lung carcinoma), 15.2 l M (nasopharyngeal carcinoma), and 8.70 l M (Leukemia) and therefore demonstrated 50% decrease in the cell growth in comparison to the vehicle.

Four fluorinated 1,2,3-triazole derivatives **194–197** (Supplementary Table S1) were reported by Kumar and coworkers by facile one pot–three component reaction employing click approach (Kumar D. et al., 2011). **194–197** were screened for inhibitory potency against Src kinase. With IC_50_ = 32–43 l M **196** was found to be the most potent inhibitory agent.

In the same year (2011) again Kumar and coworkers reported fluorinated 1,2,3-triazole conjugate **198** (Supplementary Table S1) employing the same methodology (Kumar A. et al., 2011). **198** was screened for inhibitory activity against Src kinase and SK-Ov-3 (human ovarian adenocarcinoma), MDA-MB-361 (breast carcinoma) and HT-29 (colon adenocarcinoma). **198** was modestly active for inhibition as revealed by the IC_50_ values (5.6–29.6 l M).

Escudero and coworkers reported three fluorinated triazole analogs **199–201** (Supplementary Table S1) (Escudero-Casao et al., 2018). Using SPHK1 and SPHK2 **199–201** were found to be moderately inhibitory. The IC_50_ (half maximal inhibitory concentration) values were obtained through *in vitro* TR-FRET analysis (time-resolved fluorescence energy transfer). Using reference DMS other non-fluorinated analogs did not show inhibitory activity, however, with IC_50_ values of 30.0 µM **201** was found to be most potent against SPHK1.

Pala and coworkers synthesized a novel 1,2,3-triazole linked 2,3,5,6-tetrafluorobenzenesulfonamide **202–209** (Supplementary Table S1) through click approach (Pala et al., 2014). The novel compounds **202–209** were moderately inhibitory toward cytosolic carbonic anhydrase (CA, EC 4.2.1.1) isoforms I and II and low nanomolar/subnanomolar inhibitors of the tumor-associated hCA IX and XII isoforms. In comparison to other, fluorine-substituted analogs were most inhibitory in nature probably being more acidic.

Fluorinated 1,2,3-triazole-bearing benzamide **210–216** were synthesized by Lu and coworkers (Supplementary Table S1) through Click approach (Lu et al., 2018). Using hDHODH (Human dihydroorotate dehydrogenase) assay, the reported compounds were found to be potent hDHODH inhibitors showing modest to excellent potency along with averaged clogD7.4 value. Compound **215** with IC_50_ values of 2.1 and 1.5 µM was found to be a promising inhibitor against hDHODH and effectively suppressed proliferation of the activated PBMCs. Chromogen reduction method with 2,6-dichlorophenolindophenol (DCIP). Dihydroorotate (DHO) oxidation and coenzyme Q (CoQ) reduction was coupled with chromogen reduction. The introduction of strongly electronegative and polar groups (F or CF3) at para-position of phenyl group resulted in the enhanced activity.

Avula has reported two fluorinated 1,2,3-triazole derivatives **217–218** (Supplementary Table S1) (Avula et al. 2018). XRD confirmation of **217** was also carried out. Both compounds were checked for *in vitro* α-glucosidase enzyme inhibitory activity. SAR studies and docking studies were also performed to elucidate the active pharmacophore against this enzyme. Using reference acarbose, **218** was found almost 30 times more potent with IC_50_ value of 30.6 µM that can be attributed to the presence of fluorophenyl group. The inhibitory efficiency was decreased for **217,** giving IC_50_ value of 57.7 µM bearing both trifluorophenyl.

Giroud and coworkers reported three fluorinated 1,2,3-triazole analogs **219–221** (Supplementary Table S1) (Giroud et al., 2018). Inhibitory potency of the compounds **219–221** was measured in nanomolar range for against hCatL. The fluorophenyl group being involved in parallel-shifted π···π stacking has been demonstrated for enhanced inhibitory activity.

#### 1.1.9 Antioxidant activities

Bekirican and coworkers have reported fluorinated 1,2,4-triazole derivatives **222–223** (Supplementary Table S1) (Bekirican et al., 2016). The synthesized compounds were tested for antioxidant and radical scavenging activities using CUPRAC, ABTS, and DPPH. Compounds **222–223** showed more efficient antioxidant and scavenging activity than other non-fluorinated counterparts synthesized through the same procedure.

#### 1.1.10 Antagonistic activities

Antagonistic activity of the four fluorinated 1,2,3-triazole analogs **224–227** (Supplementary Table S1) was carried out by Yu and coworkers using P2Y14 receptor (P2Y14R) (Yu et al., 2018). SAR was also explored along with docking and molecular dynamics studies at a P2Y14R homology model. The fluorinated compounds were found to be moderately potent antagonists.

#### 1.1.11 Antimalarial activities

Havaldar and coworkers reported fluorinated 1,2,4-triazole derivatives **228–229** (Supplementary Table S1) that were tested for antimalarial activity using *Plasmodium falciparum* (Havaldar and Patil., 2008). However, **229** was found to be moderately active against strains of *Plasmodium falciparum* with IC_50_ values recorded as 1.2 µM.

#### 1.1.12 Anti-inflammatory activities

Karthikeyan and coworkers reported eight thiazole-fused-1,2,4-triazoles-bearing fluorine (groups) **230–237** (Supplementary Table S1), and they were checked for anti-inflammatory activity using carrageenan-induced rat paw edema (Karthikeyan, 2009). Compounds **232**, **233,** and **237** revealed excellent anti-inflammatory activity, while **230** was found moderately active in comparison to the reference drug indomethacin. However, other compounds were weakly potent for anti-inflammation.

Anti-inflammatory activity and the mechanism involved was studied for trifluoromethylated 1,2,4-triazole derivative **238** (Supplementary Table S1) by Tu et al. (2018). TT-TFM was used for investigation and was found to suppress the activity of macrophages as revealed from the changes in body-weight and pathological damage of colon. For the purpose of anti-inflammatory screening of **238,** LPS (100 ng/ml) and varying loadings of **238** were employed for treatment of mouse primary macrophages for 24 h. Low toxicity was observed for **238** even at the highest concentration (400 μM). Effect of **238** on TNF-α produced by LPS-treatment primary mouse macrophages or resting peritoneal macrophages was also examined for evaluation of anti-inflammation potency.

### 1.2 Structural activity relationship

Regarding the structure–activity relationships, in general, it was observed that fluorinated triazole compounds were exclusively more potent than their non-fluorinated counterparts, proving the efficacy of fluorine in pharmacological and biological activities. However, Fluorine (-F) and trifluoromethyl (-CF_3_) substitution—either directly attached to ring or aryl bearing—were found more effective than other fluorine-bearing substituents [OCF_3_ (CF_3_OH) [(CF_3_)_2_OH], CF_3_SCF_3_ (CF_3_)_2_OH [(CH_2_)nCF_3_)]. Another interesting observation was that compounds having hetero- or benzo-fused triazoles (**3–14**, Supplementary Table S1) were found biologically more effective than simple triazole-containing compounds (e.g., **25–28**, Supplementary Table S1). Furthermore, fluorinated triazoles having additional electron-withdrawing groups, for example, carbonyl, carboxyl (**35–38** and **72–74**, Supplementary Table S1), were found potentially effective than having some electron-releasing groups, for example, alkoxyl groups (**61–71**, Supplementary Table S1). Moreover, fluoro aryl group directly attached with the triazole ring (N or C) exhibited significantly enhanced biological activity than other fluoro-substituted groups.

## 2 Conclusion

In the past two decades, there is considerable increase in the number of research articles describing the possible uses triazoles in medicinal chemistry with a vivid demonstration as potential drug candidates for the scientific community. There has always been a never-ending demand for a concise profiling of potent candidates in drug designing and drug development programs. Fluorinated triazoles have gained attention because of their remarkable pharmacological and biological activities. In this mini-review, we have highlighted and summarized backbone and/or functionalized fluorinated 1,2,3- and 1,2,4-triazoles with anticancer, antibacterial, antifungal, antiviral, antimicrobial, herbicidal, inhibitory, antioxidant, antagonistic, antimalarial, and anti-inflammatory properties. We claim that no such effort has been made earlier. With our current program to search for and develop potential anticancer candidates and scaffolds, fluorinated-triazoles have been extensively studied for their cytotoxic properties. The most promising fluorinated-triazoles in this direction are fused/hybrid compounds with other heterocycles and analogs with metal-based complexes. Moreover, with the increase in drug-resistive microbes, the development of effective antimicrobial candidates is another research-focused area in which fluorinated-triazoles have been evaluated. Furthermore, there are many promising and valuable fluorine-incorporating triazoles’ analogs that can serve as potential lead candidates as antiviral agents. It is revealed that many biologically and pharmacologically active fluorine-bearing triazoles have been extensively synthesized by facile click chemistry approach.

As triazole moiety has largely simplified synthetic routes, central motif to diverse functionalization, facile fluorination methods available and potential activities, it is expected to inspire the scientific community to develop these analogs toward clinical applications.
