# Supplementary material for: Fluorinated triazoles as privileged potential candidates in drug development—focusing on their biological and pharmaceutical properties
Source: Front Chem. 2022 Aug 9;10:926723. doi: 10.3389/fchem.2022.926723 (PMC9395585; doi:10.3389/fchem.2022.926723)
Supplement: Supplementary file 1 [file Table1.pdf]

Table-S1 (Chemical structures of compounds with names and target activity)

Chemical structure	No. of compounds / derivatives	Target/Activity	Reference
**Anticancer Triazoles**			
**4-aryl-5-cyano-2H-1,2,3-triazole (1-2)** 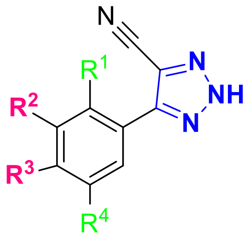	**1**: $R^1 = H$; $R^2 = H$; $R^3 = F$; $R^4 = H$ **2**: $R^1 = H$; $R^2 = F$; $R^3 =$ Pho; $R^4 = H$	Breast cancer cell line (MDA-MB-453)	(Cheng, Li et al. 2007)
**7-aryl-2-pyridyl-6,7-dihydro[1,2,4]triazolo[1,5-a][1,3,5]triazin-5-amine (3-14)** 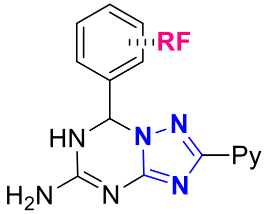	Py = 3-Py: RF = **3**: 2-F; **4**: 2-CF_3, **5**: 3-F, **6**: 3-CF_3, **7**: 4-F, **8**: 4-CF_3 Py = 4-Py, **9**: 2-F; **10**: 2-CF_3, **11**: 3-F, **12**: 3-CF_3, **13**: 4-F,**14**: 4-CF_3	Breast cancer cell line (MDA-MB-231), Colon cancer cell line (HT-29) And Lung cancer cell line A549,	(Dolzhenko, Tan et al. 2008)
**1-((2R,4S,5R)-4-hydroxy-5-(hydroxymethyl)tetrahydrofuran-2-yl)-5-(1-Aryl-1H-1,2,3-triazol-4-yl)pyrimidine-2,4(1H,3H)-dione (15-19)** 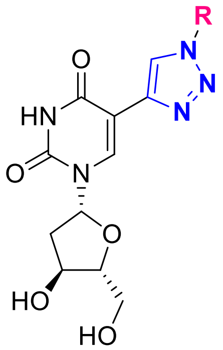	**15**: R = $-CH_2CH_2CF_2CF_3$ **16**: R = $-CH_2CH_2(CF_2)_3CF_3$ **17**: R = $-CH_2CH_2(CF_2)_5CF_3$ **18**: R = $-CH_2CH_2(CF_2)_7CF_3$ **19**: R = $-CH_2CH_2(CF_2)_9CF_3$	Human breast cancer cell line (MDA-MB-231), Renal cancer cell line (ACHN), and Prostate cancer cell line (PC-3)	Park, Yang et al. 2010)

Table-S1 (Chemical structures of compounds with names and target activity)

**3-(4-(2,4-difluorophenyl)-1H-1,2,3-triazol-1-yl)-7-(Arylperoxy)-8-methyl-2H-chromen-2-one (20-21)**<br>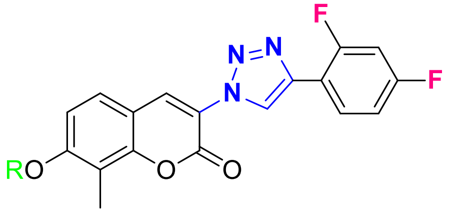	**20**: R = OH<br>**21**: R= OAc	Two breast cancer cell lines (MCF-7, SKBr-3)	(Peterson and Blagg 2010)
**2-phenyl-N-((1-(4-(trifluoromethyl)benzyl)-1H-1,2,3-triazol-4-yl)methyl)oxazole-4-carboxamide (22)**<br>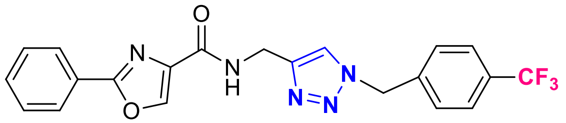	**22**	Breast cancer cell line (MCF-7), human leukemia cell line (HL-60), and Melanoma cell line (MDA-MB-435)	(Stefely, Palchaudhuri et al. 2010)
**((2R,5R)-3,4-bis(ethylthio)-5-(4-(4-fluorophenyl)-1H-1,2,3-triazol-1-yl)tetrahydrofuran-2-yl)methanol (23)**<br>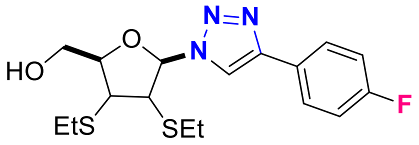	**23**	Human hepatocellular liver carcinoma cell line (HepG2)	(Yu, Wu et al. 2010)
**1-(2-(4-(4-fluorophenyl)-1H-1,2,3-triazol-1-yl)ethyl)-3-phenyl-1H-pyrazolo[3,4-d]pyrimidin-4-amine (24)**<br>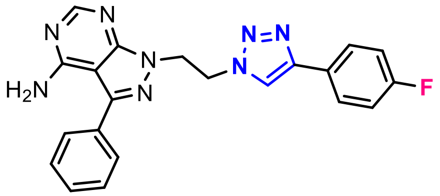	**24**	Breast cancer cell line (MDA-MB-361)	(Kumar, Ahmad et al. 2011)
**Trifluoromethylated ferrocene triazole (25-28)**<br>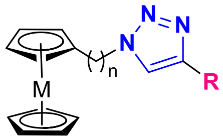	**25**: M = Fe; n = 0; R = $(CF_3)_2OH$<br>**26**: M = Fe; n = 1; R = $(CF_3)_2OH$<br>**27**: M = Fe; n = 0; R = (CH_2SCF_3)<br>**28**: M = Ru; n = 1; R = $(CF_3)_2OH$	Breast cancer cell line (MCF-7), colon cancer cell line (HT-29), and pancreas cancer cell line (PT-45)	(Maschke, Lieb et al. 2012)

Table-S1 (Chemical structures of compounds with names and target activity)

**1,3-Disubstituted-1,2,3-triazole (29-34)**<br>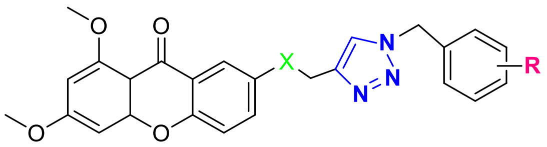	X= NH; R =<br>**29**: 2-F<br>**30**: 3-F<br>**31**: 4-F<br>X= O; R =<br>**32**: 2-F<br>**33**: 3-F<br>**34**: 4-F	Hepatoma carcinoma cell line (Bel-7402) and human cervical carcinoma cell line (HeLa)	(Zou, Zhao et al. 2012)
**Tert-butyl4-((((1-(Fluorobenzyl)-1H-1,2,3-triazol-4 yl)methyl)thio)carbonothioyl)piperazine-1-carboxylate (35-36)**<br>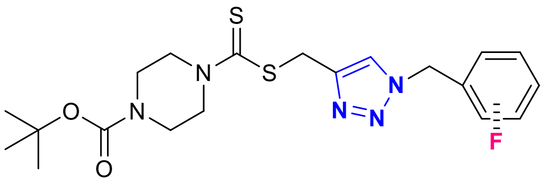	**35**: *o*-F<br>**36**: *m*-F	Human gastric cancer cell line (MGC-803) and Human breast cancer cell line (MCF-7)	(Duan, Ma et al. 2013)
**1,2,3-triazole-dithiocarbamate-urea hybrids (37-38)**<br>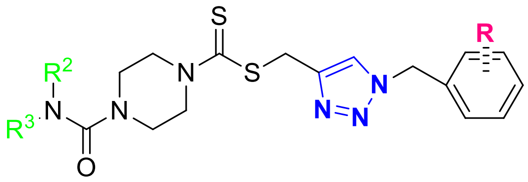	$R^2R^3NH = (CH_3)_2CHNH$<br>**37**: R^1 = *o*-F<br>**38**: R^1 = *p*-F	Human breast cancer cell line (MCF-7), Human gastric cancer cell line (MGC-803) and Hepatocellular carcinoma cell line (SMMC-7721), human esophageal cancer cell line (EC-9706)	(Duan, Zheng et al. 2013)
**6-[(N1-aryl-1H-1,2,3-triazol-4-yl)methyl]-6H-indolo[2,3-b]quinoxa-l (39-43)**	R = H, F; R^1=<br>**39**: 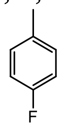<br>**40**: 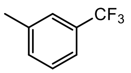	Cervical cancer cell line (HeLa), prostate cancer cell line (DU-145), and lung cancer cell line (A-549)	(Avula, Komsani et al. 2012)

Table-S1 (Chemical structures of compounds with names and target activity)

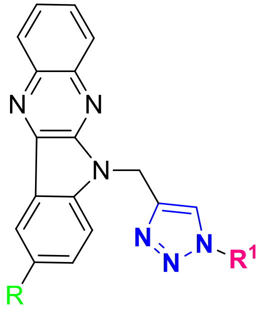	**41**: 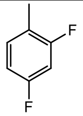<br>**42**: 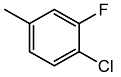		
**Novel triazole linked N-(pyrimidin-2-yl)benzo[d]thiazol-2-amine derivatives (43-50)**<br>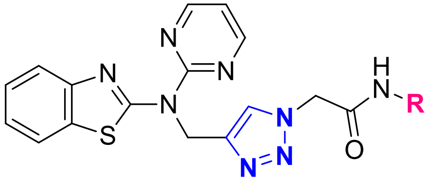	**43**: R = CH_2-4-$OCF_3C_6H_4$<br>**44**: R = 4-$SCF_3C_6H_4$<br>**45**: R = 5(CF_3)-1,3,4-Thiazole<br>**46**:R=4-F-benzothiazole morpholine<br>**47**: R = 4-FC_6H_4<br>**48**: R = 2,4-FC_6H_3<br>**49**: R = 3-$CF_3C_6H_4$<br>**50**: R = (3-Cl-4-FC_6H_3)	Lung (A549), breast (MCF-7) and skin (A375) cancer cell lines	(Kumbhare, Dadmal et al. 2015)
**1,2,3-triazole tagged pyrazolo[3,4-b]pyridine derivatives (51-56)**<br>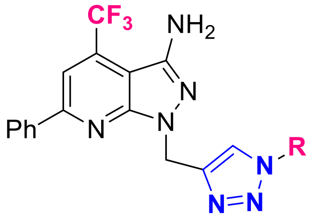	**R=**<br>**51**: C_6F_{13}-CH_2-CH_2-<br>**52**: C_8F_{17}-CH_2-CH_2-<br>**53**: 3-Cl, 4-FC_6H_3-<br>**54**: 3-$CF_3C_6H_4$-<br>**55**: 4-FC_6H_4-<br>**56**: 3-F,4-BrC_6H_3-	Human monocytic leukemia (U937), human acute monocytic leukemia (THP-1), human promyelocytic leukemia (HL-60) and Mouse Me (B16-F10) cancer cell lines	(Kurumurthy, Veeraswamy et al. 2014)
**4,5,6,7-tetrafluoro-1Aryl-benzo[d][1,2,3]triazole (57-60)**<br>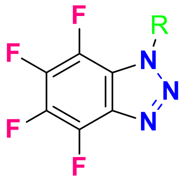	**57**: R = H<br>**58**: R = Cl<br>**59**: R = H_3CO<br>**60**: R = $(CH_3)_2N$	Hep2 (laryngeal epidermoid carcinoma) cancer cells	(Prima, Baev et al. 2017)

Table-S1 (Chemical structures of compounds with names and target activity)

**Thinopyrimidine-triazole conjugates (61-71)**<br>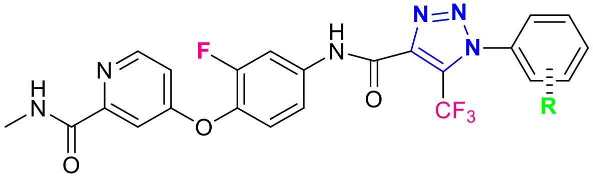<br>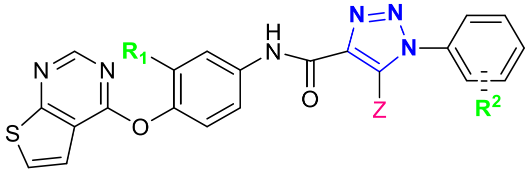	Z= CF_3; R =<br>**61**: 2-CF_3; **62**: 4-Cl; **63**: 3-F-4-F<br>**64**: 3-F-4-Cl, **65**: 4-Cl-3-CF_3<br>Z = CH_3<br>**66**: 4-F<br><br>R^1 = H; Z = CF_3; R^2 =<br>**67**: 2-CF_3<br>**68**: 4-Cl<br>**69**: 3-F-4-F<br>**70**: 2-F-4-F<br>**71**: F-F-4-Cl	Human lung cancer cell line (A549), Human liver cancer cell line (HepG2) and<br>Human breast cancer cell line (MCF-7).	(Wang, Xu et al. 2018)
**(1-((Arylphenyl]-4-yl)methyl)-1H-1,2,3-triazol-4-yl)(2-(4-Arylphenyl)imidazo[1,2-a]pyridin3yl)methanone (72-74)**<br>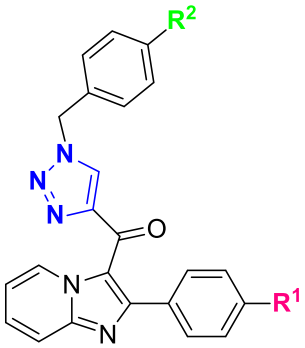	R^1 = 4-flourophenyl;<br>**72**: R^2 = OCH3<br>**73**: R^2 = H<br>**74**: R^2 = Cl	Human lung cancer (A549), Human prostate (HCT-116), and Human breast cancer cell (MDA-MB-231)	(Sayeed, Vishnuvardhan et al. 2018)
**(1aR,7aS,10aS,10bR,E)-5-((4-(3,5-bis(trifluoromethyl)phenyl)-1H-1,2,3-triazol-1-yl)methyl)-1a-methyl-8-methylene-2,3,6,7,7a,8,10a,10b-octahydrooxireno[2',3':9,10]cyclodeca[1,2-b]furan-9(1aH)-one (75)**	**75**	M9 ENL1 cell, (AML#1, and AML#2)	(Janganati, Ponder et al. 2018)

Table-S1 (Chemical structures of compounds with names and target activity)

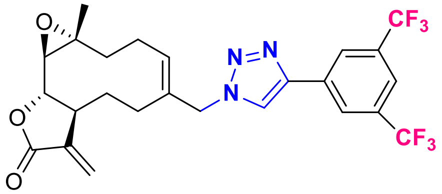			
**(4bS,7S,9aR,10S)-10-((4-(Aryl-phenyl)-1H-1,2,3-triazol-1-yl)methyl)-2,7-dihydroxy-1-methyl-8-methylene-5,6,7,8-tetrahydro-4bH-7,9a-methanobenzo[a]azulen-9(10H)-one (76-77)** 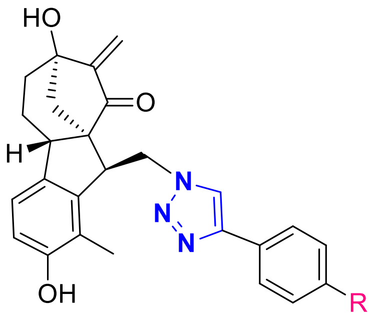	**76**: R = F<br>**77**: R = CF_3	Human myeloid leukaemia (HL-60), human lung carcinoma (A549), human liver carcinoma (SMMC-7721), human colon carcinoma (SW480) and human breast carcinoma (MCF-7) cell lines	(Wu, Wu et al. 2018)
**4-[3-fluoro-4-(morpholin-4-yl)]phenyl-1H-1,2,3-triazole (78-86)** 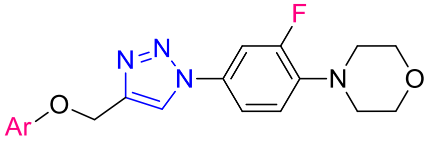	Ar =<br>**78**: C_6H_5, **79**: 2-FC_6H_4, **80**: 4-FC_6H_4 **81**: $3MeC_6H_4$, **82**: 3,5 diMeC_6H_3<br>**83**: $4MeOC_6H_4$, **84**: $3ClC_6H_4$<br>**85**: 3,5 diClC_6H_3, **86**: 4-BrC_6H_4	Breast cancer cell line (MCF-7) and cervical carcinoma cell line (HeLa)	(Narsimha, Nukala et al. 2020)
**Anti-bacterial Triazoles**			
**1-(4-Arylphenyl)-1H-1,2,3-triazole-4-carbaldehyde (87-89)** 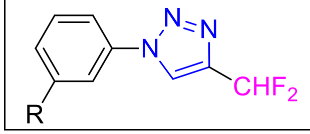	**87**: R= 4-Cl<br>**88**: R= 4-Br<br>**89**: R= 4-CH_3	Mycobacterium tuberculosis	(Costa, Boechat et al. 2006)

Table-S1 (Chemical structures of compounds with names and target activity)

**4-Aryl-7-oxo-2-(trifluoromethyl)-4,7-dihydro-1,2,4-triazolo[5,1-a]pyrimidine-6-carboxylic acid (90-94)** 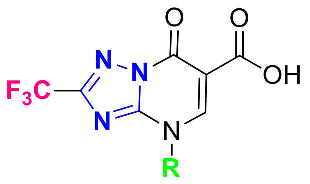	**90**: R = CH_3<br>**91**: R = C_2H_5<br>**92**: R = $-CH_2CH{=}CH_2$<br>**93**: R = $-CH_2-C_6H_4$(4-Br)<br>**94**: R = $-CH_2-C_6H_4(4-NO_2)$	Mycobacterium tuberculosis	(Abdel-Rahman, El-Koussi et al. 2009)
**2-(3-fluorophenyl)-1-((1-(Arylphenyl)-1H-1,2,3-triazol-4-yl)methyl)-1H-benzo[d]imidazole (95-100)** 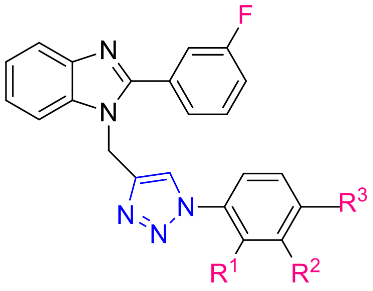	**95**: R^1 = F; R^2 = F; R^3 = F<br>**96**: R^1 = H; R^2= F; R^3 = F<br>**97**: R^1 = H; R^2 = F; R^3 = H<br>**98**: R^1 = F; R^2 = H; R^3 = Me<br>**99**: R^1 = F; R^2 = H; R^3 = F<br>**100**: R^1 = H; R^2 = H; R^3 = CF_3	(staphylococcus aureus, Pseudomonas aeruginosa) (Escherichia coli, Salmonella typhosa)	(Gill, Jadhav et al. 2008)
**3-(4-((4-(Arylphenyl)-5-thioxo-4,5-dihydro-1H-1,2,4-triazol-3-yl)methoxy)phenyl)-2-phenylquinazolin-4(3H)-one (101-102)** 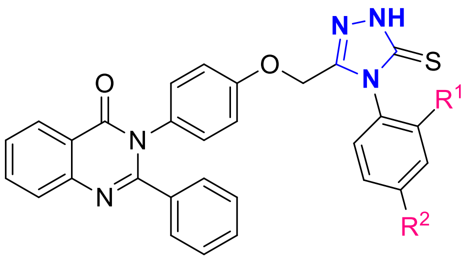	**101**: R^1 = H; R^2= F<br>**102:** R^1 = F; R^2= F	Escherichia coli. Bacillus subtilis and Staphylococcus aureus.	(Havaldar, F. et al. 2008)

Table-S1 (Chemical structures of compounds with names and target activity)

**(2-(2,4-dichloro-5-fluorophenyl)-6-(aryl)thiazolo[3,2-b][1,2,4]triazole) (103-110)** 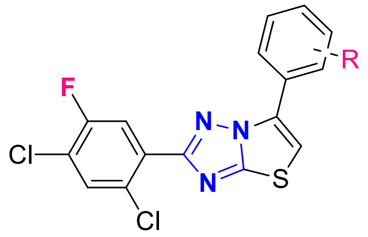	**103**: R = 4-OCH_3<br>**104**: R = 4-CH_3<br>**105**: R = 4-F<br>**106**: R = 4-Cl<br>**107**: R = 4-Br<br>**108**: R = 4-NO_2<br>**109**: R = 2,4-Cl2<br>**110**: R = 2,4-Cl2-5-F	Escherichia coli, Staphylococcus aureus, Pseudomonas aeruginosa, Streptococcus pyogenes and Klebsiella pneumoniae	(Karthikeyan 2009)
**7-((1-(Aryl)-1H-1,2,3-triazol-4-yl)methyl)-4,7-dihydro-3H-pyrrolo[2,3-d]pyrimidine (111-112)** 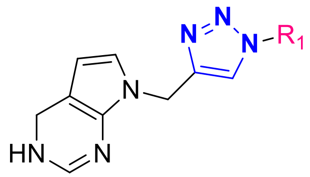	**111**: R^1 = *o*-F-Ph,<br>**112**: R^1 = *p*-F-Ph	Mycobacterium tuberculosis	(Shiva Raju, AnkiReddy et al. 2019)
**2-Aryl-3-((1-(Aryl)-1H-1,2,3-triazol-4-yl)methyl) quinazolin-4(3H)-one (113-118)** 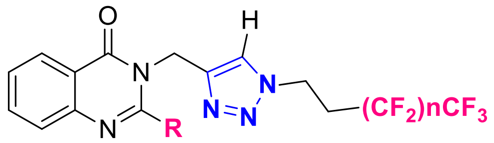	**113**: R = C_6H_5; n = 5<br>**114**: R = C_6H_5; n = 7<br>**115**: R = 2,6-$C_6H_3F_2$; n = 5<br>**116**: R = 2,6-$C_6H_3F_2$; n = 7<br>**117**: R = CF_3; n = 5<br>**118**: R = CF_3; n = 7	Gram positive bacteria (Bacillus subtilis, Staphylococcus aureus, Staphylococcus epidermidis) and gram negative bacteria (Pseudomonas aeruginosa, Escherichia coli)	(Mani Chandrika, Yakaiah et al. 2010)

Table-S1 (Chemical structures of compounds with names and target activity)

**3-(4-fluorophenyl)-3H-[1,2,3]triazolo[4,5-b]pyridine (119)** 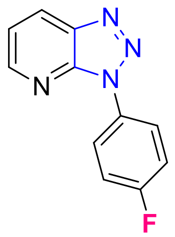	**119**	Bacillus subtilis and Escherichia coli	(Marepu, Yeturu et al. 2018)
**3-(4-fluorophenyl)-4-(prop-2-yn-1-yl)-5-(prop-2-yn-1-ylthio)-4H-1,2,4-triazole (120)** 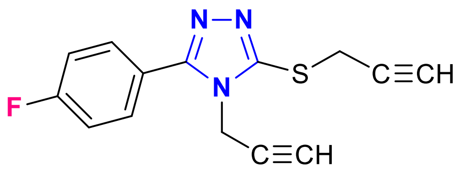	**120**	Bacillus subtilis, Streptococcus pneumonia, Staphylococcus aureus, Escherichia coli, Pseudomonas aeruginosa, and Klebsiella pneumonia	(Rezki, Mayaba et al. 2016)
**1-benzhydryl-4-((5-(2-chloro-6-fluorobenzyl)-1H-1,2,3-triazol-1-yl)methyl)piperazine (121)** 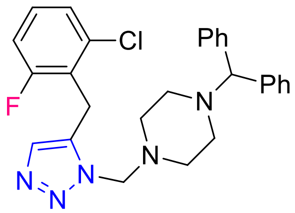	**121**	Staphylococcus aureus and Escherichia coli.	(Govindaiah, Sreenivasa et al. 2018)
**4-(2-fluoro-4-(4-([2-Aryloxy]methyl)-1H-1,2,3-triazol-1-yl)phenyl)morpholine (122-123)** 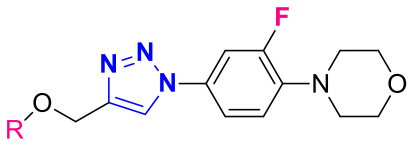	**122**: R = 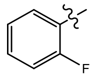<br>**123**: R = 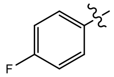	Bacillus subtilis, Staphylococcus aureus, and Staphylococcus epidermidis	(Narsimha, Nukala et al. 2020)

Table-S1 (Chemical structures of compounds with names and target activity)

**5-fluoro-1-((1-(4-fluorophenyl)-1H-1,2,3-triazol-4-yl)methyl)indoline-2,3-dione (124)** 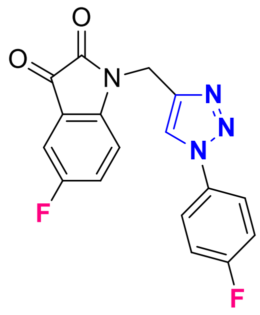	**124**	Staphylococcus epidermidis, Bacillus subtilis, Escherichia coli, and Pseudomonas aeruginosa	(Deswal, Naveen et al. 2020)
**4-amino-5-(4-fluoro-3-phenoxyphenyl)-4H-1,2,4-triazole-3-thiol (125)** 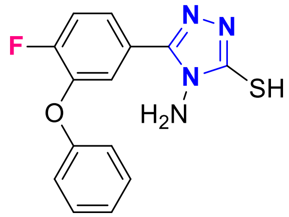	**125**	Mycobacterium tuberculosis	(Venugopala, Kandeel et al. 2020)
**3-(Aryl-phenyl)-4-(Aryl)-1H-1,2,4-triazole-5(4H)-thione (126-131)** 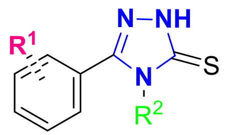	R^1 = *o*-F, R^2 = **126**: 1-naph<br>**127**: *m*-tol<br>**128***:* *p*-tol<br>R^1 = *o*-F, *m*-F<br>**129**: R^2 = 1-naph<br>**130**: R^2 = *m*-tol<br>**131**: R^2 = *p*-tol	Staphylococcus aureus,	(Kosikowska, Wujec et al. 2020)

Table-S1 (Chemical structures of compounds with names and target activity)

**3-Aryl-1-(3-fluoro-4-methylphenyl)-7Aryl-[1,2,4]triazolo[4,3-a]pyrimidin-5(1H)-one (132-135)** 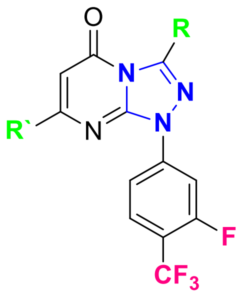	R/R*:<br>**132**: a = $COCH_3/CH_3$<br>**133**: b = COOEt $/CH_3$<br>**134**: c = $COCH_3$/Ph<br>**135**: d = COOE/Ph	Bacillus subtilis and Escherichia coli	(Muhammad, Farghaly et al. 2021)
**Antifungal Triazoles**			
**(2-(2,4-dichloro-5-fluorophenyl)-6-(aryl)thiazolo[3,2-b][1,2,4]triazole) (136-143)** 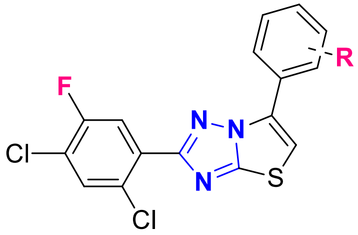	**136**: R = 4-OCH_3<br>**137**: R = 4-CH_3<br>**138**: R = 4-F<br>**139**: R = 4-Cl<br>**140**: R = 4-Br<br>**141**: R = 4-NO_2<br>**142**: R = 2,4-Cl_2<br>**143**: R = 2,4-Cl_2-5-F	Aspergillus niger, A. fumigstus, Candida albicansa, Penicillium marneffei, and Trichophyton mentagrophytes	(Karthikeyan 2009)
**3-(4-fluorophenyl)-3H-[1,2,3]triazolo[4,5-b]pyridine (144)** 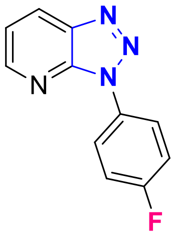	**144**	f.sp.Lycopersici. Fusarium oxysporium and Fusarium ricini	(Marepu, Yeturu et al. 2018)
**2-Aryl-3-((1-(Aryl)-1H-1,2,3-triazol-4-yl)methyl) quinazolin-4(3H)-one (145-150)**	**145**: R = C_6H_5; n = 5<br>**146**: R = C_6H_5; n = 7<br>**147**: R = 2,6-$C_6H_3F_2$; n = 5<br>**148**: R = 2,6-$C_6H_3F_2$; n = 7<br>**149**: R = CF_3; n = 5	Candida albicans, Saccharomyces cerevisiae and filamentous fungal culture like	(Mani Chandrika, Yakaiah et al. 2010)

Table-S1 (Chemical structures of compounds with names and target activity)

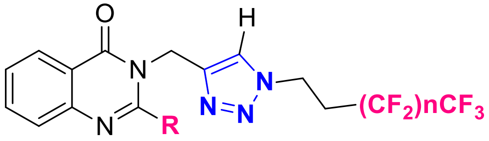	**150**: R = CF_3; n = 7	Rhizopus oryzae, Aspergillus flavus and Candida rugosa	
**2-(4-fluoro-2-(4-fluorophenoxy)phenyl)-1-(1H-1,2,4-triazol-1-yl)propan-2-ol (151)** 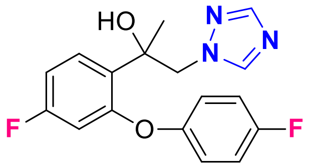	**151**	Gibberella zeae, Alternaria solani, Fusarium oxysporium, Physalospora pirco	(Yang, Zhai et al. 2015)
**N-(1-((4-substituted piper112 azin-1-yl)methyl)-3-methyl-5-thioxo-1H-1,2,4-triazol-4(5H)-yl)-2/3-fluorobenzamide (152-155)** 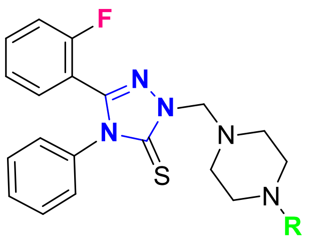	R= **152**: 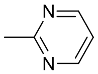 **153**: 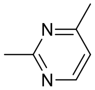 **154**: 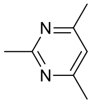 **155**: 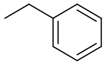	Cercospora arachidicola, Physalospora piricola, Rhizoctonia cerealis	(Zhang, Wang et al. 2016)
**(1-(Aryl)-1H-1,2,3-triazol-4-yl)methyl 4-(trifluoromethyl)benzoate (156-157)** 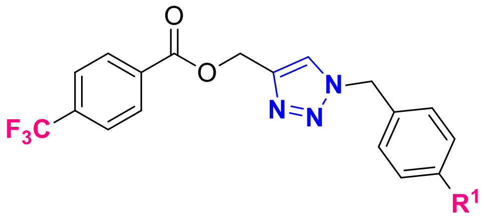	**156**: R^1 = 4-F<br>**157**: R^1 = 4-F-C_6H_5	Aspergillus niger and Candida. albicans	(Deswal, Tittal et al. 2019)

Table-S1 (Chemical structures of compounds with names and target activity)

**5-fluoro-1-((1-(4-fluorophenyl)-1H-1,2,3-triazol-4-yl)methyl)indoline-2,3-dione (158)** 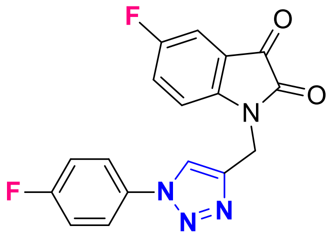	**158**	Aspergillus niger, And Candida albicans,	(Deswal, Naveen et al. 2020)
**Antiproliferative Triazoles**			
**Trifluoromethane-3,5-disubstituted 1,2,4-triazoles (159-162)** 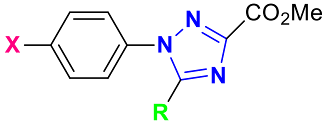	**159**: X = p-F; R = Me<br>**160**: X = p-CF_3; R = Me<br>**161**: X = m-CF_3; R = Me<br>**162**: X = p-F; R = Et	NPC-TW01 And T-cell leukemia cell	(Wang, Tseng et al. 2011)
**Antiviral Triazoles**			
**4-amino-1-((2R,3S,4R,5R)-3-fluoro-4-hydroxy-5-(hydroxymethyl)-5-(1H-1,2,3-triazol-1yl)tetrahydrofuran-2-yl)pyrimidin-2(1H)-one (163)** 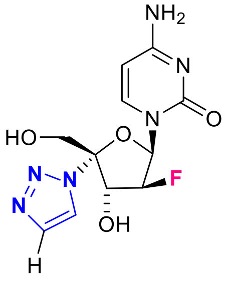	**163**	293T cell line	(Wu, Yu et al. 2013)

Table-S1 (Chemical structures of compounds with names and target activity)

**2-(trans-3-Chlorotetrahydrofuran-2-yl)-4-tosyl-5-(trifluoromethyl)-2H1,2,3-triazole (164)**<br>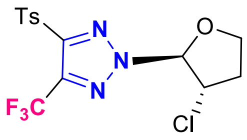	**164**	HSV-1, HSV-2 and HAdV5	(Biliavska, I., et al. 2017)
**1-((2R,3S,4R,5R)-5-azido-3-fluoro-4-hydroxy-5-(hydroxymethyl) tetrahydrofuran-2-yl)-1H-1,2,3-triazole-4-carboxamide (165)**<br>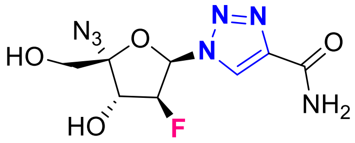	**165**	Wild-type HBV	(Liu, Peng et al. 2018)
**Ethyl7-benzyl-1-(Aryl)-5-phenyl-4,5,6,7-tetrahydro-1H-[1,2,3]triazolo[4,5-c]pyridine-7-carboxylate (166-167)**<br>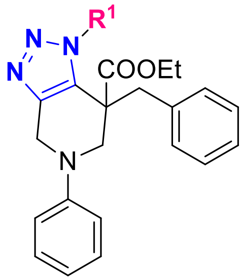	**166**: R^1 = 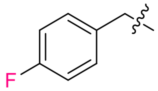<br>**167**: R^1 = 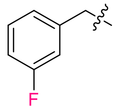	Coronavirus (229E)	(Karypidou, Ribone et al. 2018)
**Antimicrobial Triazoles**			
**2-(3-fluorophenyl)-1-((1-(Arylphenyl)-1H-1,2,3-triazol-4-yl)methyl)-1H-benzo[d]imidazole(168-173)**	**168**: R^1 = F; R^2 = F; R^3 = F<br>**169**: R^1 = H; R^2 = F; R^3 = F<br>**170**: R^1 = H; R^2 = F; R^3 = H<br>**171**: R^1 = F; R^2 = H; R^3 = Me<br>**172**: R^1 = F; R^2 = H; R^3 = F<br>**173**: R^1 = H; R^2 = H; R^3 = CF_3	Staphylococcus aureus, Pseudomonas aeruginosa, Escherichia coli and Salmonella typhosa.	(Gill, Jadhav et al. 2008)

Table-S1 (Chemical structures of compounds with names and target activity)

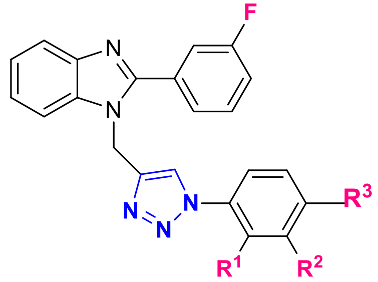			
**N-di(trifluoroacetyl)sulfonamide (174-177)** 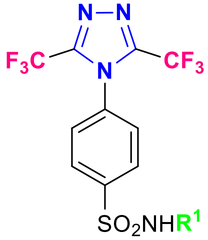	**174**: R^1 = H,<br>**175**: R^1 = CH_3CO,<br>**176**: R^1 = 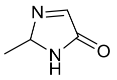 **177**: R^1 = 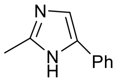	Staphylococcus aureus, Escherichia coli, Aspergillus niger and Candida albicans	(Faidallah, Khan et al. 2011)
**(E)-3-(2-((1-(4-fluorophenyl)-1H-1,2,3-triazol-4-yl)methoxy) naphthalen-1-yl)-1-(4-methoxyphenyl)prop-2-en-1-one (178)** 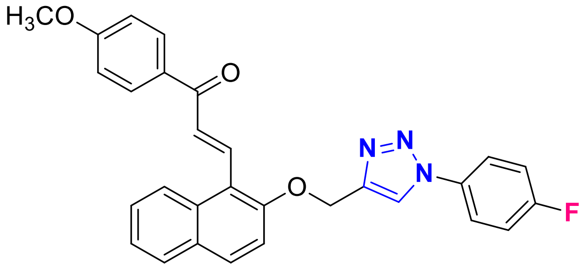	**178**	Staphylococcus epidermidis, Bacillus subtilis, Escherichia. coli, Pseudomonas. aeruginosa, Aspergillus niger and Candida albicans	(Yadav, Lal et al. 2018)
**Herbicidal Triazoles**			

Table-S1 (Chemical structures of compounds with names and target activity)

**(E)-N-(argiomethylene)-3-(trifluoromethyl)-5-(3-(trifluoromethyl)phenoxy)-4H-1,2,4-triazol-4-amine (179)** 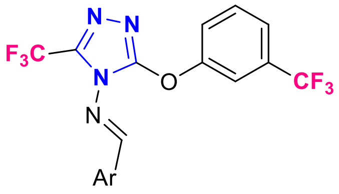	**179**	Brassica campestris, and Echinochloa crus-galli	(Zhang and Shi 2014)
**Aryl-N-(3-((Arylbenzyl)thio)-4H-1,2,4-triazol-4-yl)benzamide (180-185)** 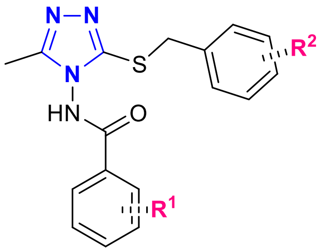	R^1 = *o*-F;<br>**180**: R^2 = *o*-F<br>**181**: R^2 = *m*-F<br>**182**: R^2 = *p*-F<br>R^1 = *m*-F;<br>**183**: R^2 = *o*-F<br>**184**: R^2 = *m*-F<br>**185**: R^2 = *p*-F	Brassica campestris, and Echinochloa crusgalli	(Liu, Weng et al. 2013)
**N-(1-((4-substituted piper112 azin-1-yl)methyl)-3-methyl-5-thioxo-1H-1,2,4-triazol-4(5H)-yl)-2/3-fluorobenzamide (186-189)** 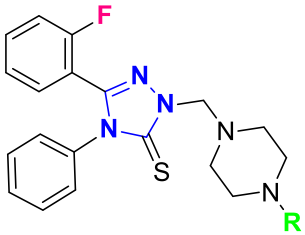	R =<br>**186**: 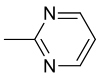 **187**: 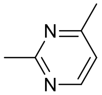 **188**: 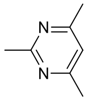 **189**: 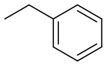	Brassica campestris and Echinochloa crus-galli	(Zhang, Wang et al. 2016)
**Inhibitory Triazoles**			

Table-S1 (Chemical structures of compounds with names and target activity)

**Trifluoromethane-3,5-disubstituted 1,2,4-triazoles (190-193)**<br>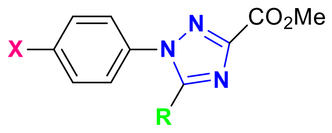	**190**: X = *p*-F; R = Me<br>**191**: X = *p*-CF_3; R = Me<br>**192**: X = *m*-CF_3; R = Me<br>**193**: X = *p*-F; R = Et	Lung carcinoma, Nasopharyngeal, And T-cell leukemia (Jurkat) cells	(Wang, Tseng et al. 2011)
**2-(4-(Aryl)-1H-1,2,3-triazol-1-yl)-1-phenylethanone (194-197)**<br>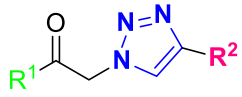	**194**; $R^1 = C_6H_5$; R^2= 4-F-3-$CH_3C_6H_3$<br>**195**:R^1=4-$CH_3C_6H_4$;R^2=4-F-3-$CH_3C_6H_3$<br>**196**: R^1 = 4-ClC_6H_4; R^2= 4-FC_6H_4<br>**197**: R^1 = 4-BrC_6H_4; R^2= 4-FC_6H_4	Src kinase	(Kumar, Reddy et al. 2011)
**1-(2-(4-(4-fluorophenyl)-1H-1,2,3-triazol-1-yl)ethyl)-3-phenyl-1H-pyrazolo[3,4-d]pyrimidin-4amine (198)**<br>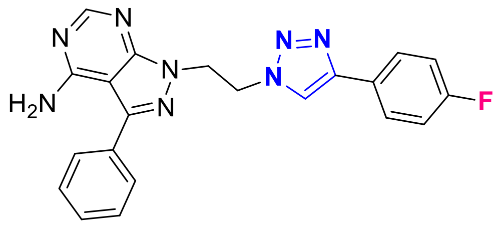	**198**	Src kinase	(Kumar, Ahmad et al. 2011)
**Tert-butyl((1S,2S)-1,3-dihydroxy-1-(1-Aryl-1H-1,2,3-triazol-4-yl)propan-2-yl) (199-201)**<br>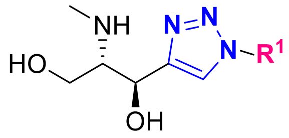	**199**: R^1; $C_{11}H_{22}CF_3$ 61%<br>**200**: R^1; $C_{10}H_{20}CF_2CF_3$ 63%<br>**201**: R^1; $C_9H_{18}CF_2CF_2CF_3$ 69%	SPHK1 and SPHK2	(Escudero-Casao, Cardona et al. 2018)
**2,3,5,6-tetrafluoro-4-(4-Aryl-1H-1,2,3-triazol-1-yl)benzenesulfinamide (202-209)**	R =<br>**202**: C_6H_5<br>**203**: $BrCH_2CH_2$<br>**204**: MeOOC<br>**205**: *p*-Me-C_6H_4<br>**206**: $Cl(CH_2)_3$	Isoform hCA I, II, IX and XII	(Pala, Micheletto et al. 2014)

Table-S1 (Chemical structures of compounds with names and target activity)

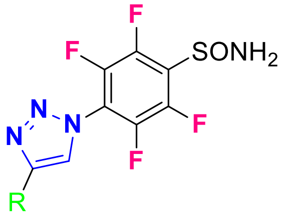	**207**: $Cl(CH_2)_4$<br>**208**: $HO\text{-}CH_2$<br>**209**: $H2N\text{-}CH_2$		
**2-(4-((Aryl-phenoxy)methyl)-Aryl-1,2,3-triazol-1-yl)benzamide(210-216)**<br>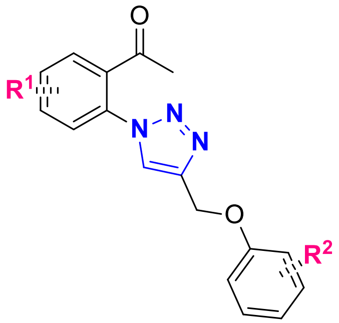	R^1= H; R^2 =<br>**210**: 4-F<br>**211**: 4-CF_3<br>**212**: 3-CF_3<br>**213**: 3,4-2F<br>R^1= 4,5-2F; R^2 =<br>**214**: 4-F<br>**215**: 4-CF_3<br>**216**: 3,4-2F	Hdhodh	(Lu, Cai et al. 2018)
**(R)-1-(2-(4-bromo-2-methoxyphenoxy)propyl)-4-(Aryll)-4-yl)-1H-1,2,3-triazole (217-218)**<br>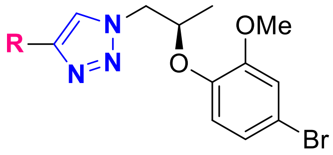	**217**: R = 2-$CF_3C_6H_4$<br>**218**: R = 4-FC_6H_4	α-glucosidase enzyme	(Avula, Khan et al. 2018)
**(S)-N-(3-(3-chloro-4-(trifluoromethoxy)phenyl)-1-((1-cyanocyclopropyl)amino)-1-oxopropan-2-yl)-2-(Aryl)-2H-1,2,3triazole-4-carboxamide (219-221)**	R=<br>**219**: 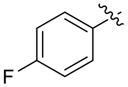<br>**220**: 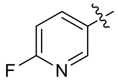<br>**221**: 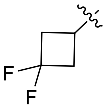	Human cathepsin L (hCatL)	(Giroud, Kuhn et al. 2018)

Table-S1 (Chemical structures of compounds with names and target activity)

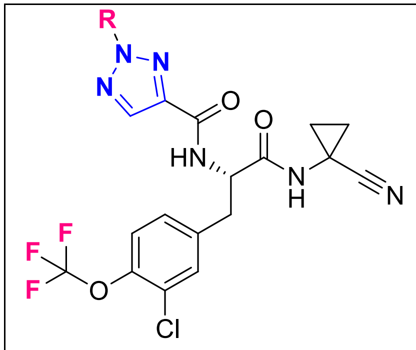			
**Antioxidant Triazoles**			
**4-amino-3-(3/4-fluorobenzyl)-1-((4-(4-fluorophenyl)-5-thioxo-4,5-dihydro-1H-1,2,4-triazol-3-yl)methyl)-1H-1,2,4-triazol-5(4H)-one (222-223)** 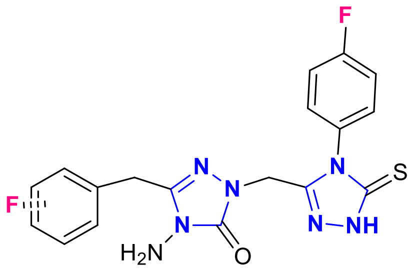	**222**: *m*-F<br>**223**: *p*-F	Jack bean urease	(Bekirican, O. et al. 2016)
**Antagonistic Triazoles**			
**3-(Aryl)-5-(4-(4-(trifluoromethyl)phenyl)-1H-1,2,3-triazol-1-yl)benzoic acid (224-227)** 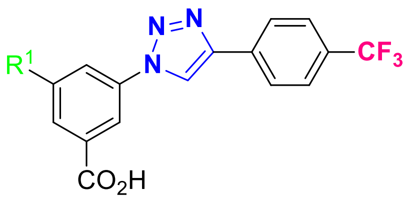	R^1 =<br>**224**: -OH<br>**225**: 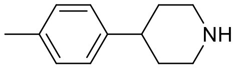<br>**226**: 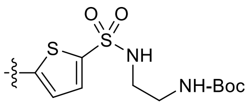	P2Y14R	(Yu, Ciancetta et al. 2018)

Table-S1 (Chemical structures of compounds with names and target activity)

	**227**: 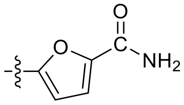		
**Antimalarial Triazoles**			
**3-(4-((4-(Arylphenyl)-5-thioxo-4,5-dihydro-1H-1,2,4-triazol-3-yl)methoxy)phenyl)-2-phenylquinazolin-4(3H)-one (228-229)** 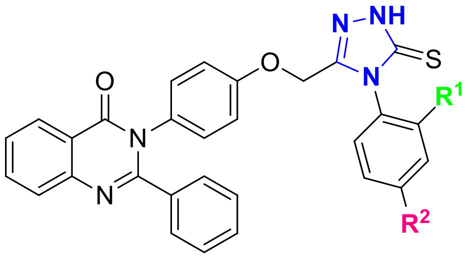	**228**: R^1 = H; R^2 = F<br>**229**: R^1 = F; R^2 = F	Plasmodium falciparum	(Havaldar, F. et al. 2008)
**Anti-inflammatory Triazoles**			
**(2-(2,4-dichloro-5-fluorophenyl)-6-(aryl)thiazolo[3,2-b][1,2,4]triazole) (230 – 237)** 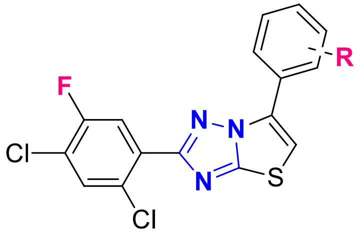	R =<br>**230**: 4-OCH_3<br>**231**: 4-CH_3<br>**232**: 4-F<br>**233**: 4-Cl<br>**234**: 4-Br<br>**235**: 4-NO_2<br>**236**: 2,4-Cl_2<br>**237**: 2,4-Cl_2-5-F	*Escherichia coli*, *Staphylococcus aureus*, *Aspergillus niger* and *Candida albicans*	(Karthikeyan 2009)
**(R)-2,2,2-trifluoro-1-(6-phenylthiazolo[3,2-b][1,2,4]triazol-5-yl)ethanamine (238)**	**238**	TT-TFM	(Tu, Yu et al. 2018)

Table-S1 (Chemical structures of compounds with names and target activity)

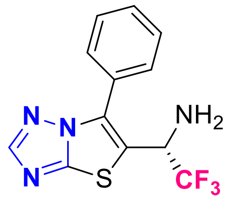			

"<Antimalarial activity-1.pdf>."
"<Antioxidant activity-1.pdf>."
Abdel-Rahman, H. M., N. A. El-Koussi and H. Y. Hassan (2009). "Fluorinated 1,2,4-Triazolo[1,5-a]pyrimidine-6-carboxylic acid derivatives as antimycobacterial agents." Arch Pharm (Weinheim) **342**(2): 94-99.
Avula, S., J. R. Komsani, S. Koppireddi, R. Yadla, A. K. Kanugula and S. Kotamraju (2012). "Synthesis and cytotoxicity of novel 6H-indolo[2,3-b]quinoxaline derivatives." Medicinal Chemistry Research **22**(8): 3712-3718.
Avula, S. K., A. Khan, N. U. Rehman, M. U. Anwar, Z. Al-Abri, A. Wadood, M. Riaz, R. Csuk and A. Al-Harrasi (2018). "Synthesis of 1H-1,2,3-triazole derivatives as new alpha-glucosidase inhibitors and their molecular docking studies." Bioorg Chem **81**: 98-106.
Cheng, Z. Y., W. J. Li, F. He, J. M. Zhou and X. F. Zhu (2007). "Synthesis and biological evaluation of 4-aryl-5-cyano-2H-1,2,3-triazoles as inhibitor of HER2 tyrosine kinase." Bioorg Med Chem **15**(3): 1533-1538.
Costa, M. S., N. Boechat, E. A. Rangel, C. da Silva Fde, A. M. de Souza, C. R. Rodrigues, H. C. Castro, I. N. Junior, M. C. Lourenco, S. M. Wardell and V. F. Ferreira (2006). "Synthesis, tuberculosis inhibitory activity, and SAR study of N-substituted-phenyl-1,2,3-triazole derivatives." Bioorg Med Chem **14**(24): 8644-8653.
Deswal, S., Naveen, R. K. Tittal, D. Ghule Vikas, K. Lal and A. Kumar (2020). "5-Fluoro-1H-indole-2,3-dione-triazoles- synthesis, biological activity, molecular docking, and DFT study." Journal of Molecular Structure **1209**.
Deswal, S., R. K. Tittal, P. Yadav, K. Lal, G. Vikas D and N. Kumar (2019). "Cellulose-Supported CuI-Nanoparticles-Mediated Green Synthesis of Trifluoromethylbenzoate-Linked Triazoles for Pharmacological & DFT study." ChemistrySelect **4**(2): 759-764.
Dolzhenko, A. V., B. J. Tan, A. V. Dolzhenko, G. N. C. Chiu and W. K. Chui (2008). "Synthesis and biological activity of fluorinated 7-aryl-2-pyridyl-6,7-dihydro[1,2,4]triazolo[1,5-a][1,3,5]triazin-5-amines." Journal of Fluorine Chemistry **129**(5): 429-434.
Duan, Y. C., Y. C. Ma, E. Zhang, X. J. Shi, M. M. Wang, X. W. Ye and H. M. Liu (2013). "Design and synthesis of novel 1,2,3-triazole-dithiocarbamate hybrids as potential anticancer agents." Eur J Med Chem **62**: 11-19.
Duan, Y. C., Y. C. Zheng, X. C. Li, M. M. Wang, X. W. Ye, Y. Y. Guan, G. Z. Liu, J. X. Zheng and H. M. Liu (2013). "Design, synthesis and antiproliferative activity studies of novel 1,2,3-triazole-dithiocarbamate-urea hybrids." Eur J Med Chem **64**: 99-110.
Escudero-Casao, M., A. Cardona, R. Beltran-Debon, Y. Diaz, M. I. Matheu and S. Castillon (2018). "Fluorinated triazole-containing sphingosine analogues. Syntheses and in vitro evaluation as SPHK inhibitors." Org Biomol Chem **16**(39): 7230-7235.
Faidallah, H. M., K. A. Khan and A. M. Asiri (2011). "Synthesis and biological evaluation of new 3,5-di(trifluoromethyl)-1,2,4-triazolesulfonylurea and thiourea derivatives as antidiabetic and antimicrobial agents." Journal of Fluorine Chemistry **132**(11): 870-877.
Gill, C., G. Jadhav, M. Shaikh, R. Kale, A. Ghawalkar, D. Nagargoje and M. Shiradkar (2008). "Clubbed [1,2,3] triazoles by fluorine benzimidazole: a novel approach to H37Rv inhibitors as a potential treatment for tuberculosis." Bioorg Med Chem Lett **18**(23): 6244-6247.

Table-S1 (Chemical structures of compounds with names and target activity)

Giroud, M., B. Kuhn, S. Saint-Auret, C. Kuratli, R. E. Martin, F. Schuler, F. Diederich, M. Kaiser, R. Brun, T. Schirmeister and W. Haap (2018). "2 H-1,2,3-Triazole-Based Dipeptidyl Nitriles: Potent, Selective, and Trypanocidal Rhodesain Inhibitors by Structure-Based Design." J Med Chem **61**(8): 3370-3388.

Govindaiah, S., S. Sreenivasa, R. A. Ramakrishna, T. M. C. Rao and H. Nagabhushana (2018). "Regioselective Synthesis, Antibacterial, Molecular Docking and Fingerprint Applications of 1-Benzhydrylpiperazine Derivatized 1,4-Disubstituted 1,2,3-Triazoles." ChemistrySelect **3**(28): 8111-8117.

Janganati, V., J. Ponder, M. Balasubramaniam, P. Bhat-Nakshatri, E. E. Bar, H. Nakshatri, C. T. Jordan and P. A. Crooks (2018). "MMB triazole analogs are potent NF-kappaB inhibitors and anti-cancer agents against both hematological and solid tumor cells." Eur J Med Chem **157**: 562-581.

Karthikeyan, M. S. (2009). "Synthesis, analgesic, anti-inflammatory and antimicrobial studies of 2,4-dichloro-5-fluorophenyl containing thiazolotriazoles." Eur J Med Chem **44**(2): 827-833.

Karypidou, K., S. R. Ribone, M. A. Quevedo, L. Persoons, C. Pannecouque, C. Helsen, F. Claessens and W. Dehaen (2018). "Synthesis, biological evaluation and molecular modeling of a novel series of fused 1,2,3-triazoles as potential anti-coronavirus agents." Bioorg Med Chem Lett **28**(21): 3472-3476.

Kosikowska, U., M. Wujec, N. Trotsko, W. Plonka, P. Paneth and A. Paneth (2020). "Antibacterial Activity of Fluorobenzoylthiosemicarbazides and Their Cyclic Analogues with 1,2,4-Triazole Scaffold." Molecules **26**(1).

Kumar, A., I. Ahmad, B. S. Chhikara, R. Tiwari, D. Mandal and K. Parang (2011). "Synthesis of 3-phenylpyrazolopyrimidine-1,2,3-triazole conjugates and evaluation of their Src kinase inhibitory and anticancer activities." Bioorg Med Chem Lett **21**(5): 1342-1346.

Kumar, D., V. B. Reddy, A. Kumar, D. Mandal, R. Tiwari and K. Parang (2011). "Click chemistry inspired one-pot synthesis of 1,4-disubstituted 1,2,3-triazoles and their Src kinase inhibitory activity." Bioorg Med Chem Lett **21**(1): 449-452.

Kumbhare, R. M., T. L. Dadmal, M. J. Ramaiah, K. S. Kishore, S. N. Pushpa Valli, S. K. Tiwari, K. Appalanaidu, Y. K. Rao and M. P. Bhadra (2015). "Synthesis and anticancer evaluation of novel triazole linked N-(pyrimidin-2-yl)benzo[d]thiazol-2-amine derivatives as inhibitors of cell survival proteins and inducers of apoptosis in MCF-7 breast cancer cells." Bioorg Med Chem Lett **25**(3): 654-658.

Kurumurthy, C., B. Veeraswamy, P. Sambasiva Rao, G. Santhosh Kumar, P. Shanthan Rao, V. Loka Reddy, J. Venkateswara Rao and B. Narsaiah (2014). "Synthesis of novel 1,2,3-triazole tagged pyrazolo[3,4-b]pyridine derivatives and their cytotoxic activity." Bioorg Med Chem Lett **24**(3): 746-749.

Liu, X.-H., J.-Q. Weng, B.-L. Wang, Y.-H. Li, C.-X. Tan and Z.-M. Li (2013). "Microwave-assisted synthesis of novel fluorinated 1,2,4-triazole derivatives, and study of their biological activity." Research on Chemical Intermediates **40**(8): 2605-2612.

Liu, Y., Y. Peng, J. Lu, J. Wang, H. Ma, C. Song, B. Liu, Y. Qiao, W. Yu, J. Wu and J. Chang (2018). "Design, synthesis, and biological evaluation of new 1,2,3-triazolo-2'-deoxy-2'-fluoro- 4'-azido nucleoside derivatives as potent anti-HBV agents." Eur J Med Chem **143**: 137-149.

Lu, K., L. Cai, X. Zhang, G. Wu, C. Xu, Y. Zhao and P. Gong (2018). "Design, synthesis, and biological evaluation of novel substituted benzamide derivatives bearing a 1,2,3-triazole moiety as potent human dihydroorotate dehydrogenase inhibitors." Bioorg Chem **76**: 528-537.

Mani Chandrika, P., T. Yakaiah, G. Gayatri, K. Pranay Kumar, B. Narsaiah, U. S. Murthy and A. Raghu Ram Rao (2010). "Click chemistry: studies on the synthesis of novel fluorous tagged triazol-4-yl substituted quinazoline derivatives and their biological evaluation--theoretical and experimental validation." Eur J Med Chem **45**(1): 78-84.

Marepu, N., S. Yeturu and M. Pal (2018). "1,2,3-Triazole fused with pyridine/pyrimidine as new template for antimicrobial agents: Regioselective synthesis and identification of potent N-heteroarenes." Bioorg Med Chem Lett **28**(20): 3302-3306.

Maschke, M., M. Lieb and N. Metzler-Nolte (2012). "Biologically Active Trifluoromethyl-Substituted Metallocene Triazoles: Characterization, Electrochemistry, Lipophilicity, and Cytotoxicity." European Journal of Inorganic Chemistry **2012**(36): 5953-5959.

Muhammad, Z. A., T. A. Farghaly, I. Althagafi, S. A. Al-Hussain, M. E. A. Zaki and M. F. Harras (2021). "Synthesis of antimicrobial azoloazines and molecular docking for inhibiting COVID-19." J Heterocycl Chem **58**(6): 1286-1301.

Narsimha, S., S. K. Nukala, T. Savitha Jyostna, M. Ravinder, M. Srinivasa Rao and N. Vasudeva Reddy (2020). "One-pot synthesis and biological evaluation of novel 4-[3-fluoro-4-(morpholin-4-yl)]phenyl-1

Table-S1 (Chemical structures of compounds with names and target activity)

H

-1,2,3-triazole derivatives as potent antibacterial and anticancer agents." Journal of Heterocyclic Chemistry **57**(4): 1655-1665.
Pala, N., L. Micheletto, M. Sechi, M. Aggarwal, F. Carta, R. McKenna and C. T. Supuran (2014). "Carbonic Anhydrase Inhibition with Benzenesulfonamides and Tetrafluorobenzenesulfonamides Obtained via Click Chemistry." ACS Med Chem Lett **5**(8): 927-930.
Peterson, L. B. and B. S. Blagg (2010). "Click chemistry to probe Hsp90: Synthesis and evaluation of a series of triazole-containing novobiocin analogues." Bioorg Med Chem Lett **20**(13): 3957-3960.
Prima, D. O., D. S. Baev, E. V. Vorontsova, T. S. Frolova, I. Y. Bagryanskaya, Y. G. Slizhov, T. G. Tolstikova, A. Y. Makarov and A. V. Zibarev (2017). New cancer cells apoptosis agents: Fluorinated aza-heterocycles.
Rezki, N., M. M. Mayaba, F. F. Al-blewi, M. R. Aouad and E. S. H. El Ashry (2016). "Click 1,4-regioselective synthesis, characterization, and antimicrobial screening of novel 1,2,3-triazoles tethering fluorinated 1,2,4-triazole and lipophilic side chain." Research on Chemical Intermediates **43**(2): 995-1011.
Sayeed, I. B., M. Vishnuvardhan, A. Nagarajan, S. Kantevari and A. Kamal (2018). "Imidazopyridine linked triazoles as tubulin inhibitors, effectively triggering apoptosis in lung cancer cell line." Bioorg Chem **80**: 714-720.
Shiva Raju, K., S. AnkiReddy, G. Sabitha, V. Siva Krishna, D. Sriram, K. Bharathi Reddy and S. Rao Sagurthi (2019). "Synthesis and biological evaluation of 1H-pyrrolo[2,3-d]pyrimidine-1,2,3-triazole derivatives as novel anti-tubercular agents." Bioorg Med Chem Lett **29**(2): 284-290.
Stefely, J. A., R. Palchaudhuri, P. A. Miller, R. J. Peterson, G. C. Moraski, P. J. Hergenrother and M. J. Miller (2010). "N-((1-benzyl-1H-1,2,3-triazol-4-yl)methyl)arylamide as a new scaffold that provides rapid access to antimicrotubule agents: synthesis and evaluation of antiproliferative activity against select cancer cell lines." J Med Chem **53**(8): 3389-3395.
Tu, T., M. Yu, Y. Zhang, X. Shi, J. Xu, J. Hu, J. Gan, W. He, L. Dong, J. Han, Z. Huang, Y. Pan and J. Zhang (2018). "A novel fluorinated triazole derivative suppresses macrophage activation and alleviates experimental colitis via a Twist1-dependent pathway." Biochem Pharmacol **155**: 275-287.
Venugopala, K. N., M. Kandeel, M. Pillay, P. K. Deb, H. H. Abdallah, M. F. Mahomoodally and D. Chopra (2020). "Anti-Tubercular Properties of 4-Amino-5-(4-Fluoro-3- Phenoxyphenyl)-4H-1,2,4-Triazole-3-Thiol and Its Schiff Bases: Computational Input and Molecular Dynamics." Antibiotics (Basel) **9**(9).
Wang, L., S. Xu, X. Liu, X. Chen, H. Xiong, S. Hou, W. Zou, Q. Tang, P. Zheng and W. Zhu (2018). "Discovery of thinopyrimidine-triazole conjugates as c-Met targeting and apoptosis inducing agents." Bioorg Chem **77**: 370-380.
Wang, L. Y., W. C. Tseng, T. S. Wu, K. Kaneko, H. Takayama, M. Kimura, W. C. Yang, J. B. Wu, S. H. Juang and F. F. Wong (2011). "Synthesis and antiproliferative evaluation of 3,5-disubstituted 1,2,4-triazoles containing flurophenyl and trifluoromethanephenyl moieties." Bioorg Med Chem Lett **21**(18): 5358-5362.
Wu, J., W. Yu, L. Fu, W. He, Y. Wang, B. Chai, C. Song and J. Chang (2013). "Design, synthesis, and biological evaluation of new 2'-deoxy-2'-fluoro-4'-triazole cytidine nucleosides as potent antiviral agents." Eur J Med Chem **63**: 739-745.
Wu, M. J., D. M. Wu, J. B. Chen, J. F. Zhao, L. Gong, Y. X. Gong, Y. Li, X. D. Yang and H. Zhang (2018). "Synthesis and anti-proliferative activity of allogibberic acid derivatives containing 1,2,3-triazole pharmacophore." Bioorg Med Chem Lett **28**(14): 2543-2549.
Yadav, P., K. Lal, L. Kumar, A. Kumar, A. Kumar, A. K. Paul and R. Kumar (2018). "Synthesis, crystal structure and antimicrobial potential of some fluorinated chalcone-1,2,3-triazole conjugates." Eur J Med Chem **155**: 263-274.
Yang, S.-H., Z.-W. Zhai and S.-W. Zhang (2015). "Synthesis and Antifungal Activities of Novel Fluorine-Containing Triazole Compounds." Asian Journal of Chemistry **27**(1): 173-176.
Yu, J., A. Ciancetta, S. Dudas, S. Duca, J. Lottermoser and K. A. Jacobson (2018). "Structure-Guided Modification of Heterocyclic Antagonists of the P2Y14 Receptor." J Med Chem **61**(11): 4860-4882.
Yu, J. L., Q. P. Wu, Q. S. Zhang, Y. H. Liu, Y. Z. Li and Z. M. Zhou (2010). "Synthesis and antitumor activity of novel 2',3'-dideoxy-2',3'-diethanethionucleosides bearing 1,2,3-triazole residues." Bioorg Med Chem Lett **20**(1): 240-243.

Table-S1 (Chemical structures of compounds with names and target activity)

Zhang, H.-Y. and D.-Q. Shi (2014). "Synthesis and Herbicidal Activities of 3-Trifluoromethyl-5-[3-(trifluoromethyl)phenoxy]-1,2,4-Triazol-4-Schiff Bases." Journal of Heterocyclic Chemistry **51**(S1): E344-E348.

Zhang, L.-Y., B.-L. Wang, Y.-Z. Zhan, Y. Zhang, X. Zhang and Z.-M. Li (2016). "Synthesis and biological activities of some fluorine- and piperazine-containing 1,2,4-triazole thione derivatives." Chinese Chemical Letters **27**(1): 163-167.

Zou, Y., Q. Zhao, H. Hu, L. Hu, S. Yu, M. Xu and Q. Wu (2012). "Synthesis and in vitro antitumor activities of xanthone derivatives containing 1,4-disubstituted-1,2,3-triazole moiety." Arch Pharm Res **35**(12): 2093-2104.
